# Environmental DNA as an innovative technique to identify the origins of falsified antimalarial tablets—a pilot study of the pharmabiome

**DOI:** 10.1038/s41598-022-25196-0

**Published:** 2022-12-20

**Authors:** Jennifer M. Young, Craig Liddicoat, Kor-jent van Dijk, Patricia Tabernero, Celine Caillet, Nicholas J. White, Adrian Linacre, Jeremy J. Austin, Paul N. Newton

**Affiliations:** 1 College of Science and Engineering, Flinders University, Adelaide, South Australia 5042 Australia; 2 School of Public Health, The University of Adelaide, Adelaide, South Australia 5005 Australia; 3 Advanced DNA, Identification and Forensic Facility, School of Biological Sciences, University of Adelaide, Adelaide, South Australia 5005 Australia; 4 Lao-Oxford-Mahosot-Hospital-Wellcome Trust Research Unit (LOMWRU), Microbiology Laboratory, Mahosot Hospital, Vientiane, Lao PDR; 5 Centre for Tropical Medicine and Global Health, Nuffield Department of Medicine, University of Oxford, Oxford, OX3 7LJ UK; 6 Infectious Diseases Data Observatory, Nuffield Department of Medicine, University of Oxford, Oxford, OX3 7LJ UK; 7 Public Health Unit, Faculty of Medicine, University of Alcalá, 28871 Alcalá de Henares, Spain; 8 Mahidol Oxford Research Unit, Faculty of Tropical Medicine, Mahidol University, Bangkok, 10400 Thailand; 9 Australian Centre for Ancient DNA, School of Biological Sciences, University of Adelaide, Adelaide, South Australia 5005 Australia; 10 Faculty of Infectious and Tropical Diseases, London School of Hygiene and Tropical Medicine, London, WC1E 7HT UK; 11 School of Public Health, Boston University, Boston, MA 02118 USA

**Keywords:** Genomics, Therapeutics

## Abstract

Falsified medicines are a major threat to global health. Antimalarial drugs have been particularly targeted by criminals. As DNA analysis has revolutionized forensic criminology, we hypothesized that these techniques could also be used to investigate the origins of falsified medicines. Medicines may contain diverse adventitious biological contamination, and the sealed nature of blister-packages may capture and preserve genetic signals from the manufacturing processes allowing identification of production source(s). We conducted a blinded pilot study to determine if such environmental DNA (eDNA) could be detected in eleven samples of falsified and genuine artesunate antimalarial tablets, collected in SE Asia, which could be indicative of origin. Massively Parallel Sequencing (MPS) was used to characterize microbial and eukaryote diversity. Two mitochondrial DNA analysis approaches were explored to detect the presence of human DNA. Trace eDNA from these low biomass samples demonstrated sample specific signals using two target markers. Significant differences in bacterial and eukaryote DNA community structures were observed between genuine and falsified tablets and between different packaging types of falsified artesunate. Human DNA, which was indicative of likely east Asian ancestry, was found in falsified tablets. This pilot study of the ‘pharmabiome’ shows the potential of environmental DNA as a powerful forensic tool to assist with the identification of the environments, and hence location and timing, of the source and manufacture of falsified medicines, establish links between seizures and complement existing tools to build a more complete picture of criminal trade routes. The finding of human DNA in tablets raises important ethical issues that need to be addressed.

## Introduction

The criminal manufacture of falsified medicinal products is a global but neglected public health issue^1^. Overall, it has been estimated that in low and middle-income countries some 10% of medicines are either substandard or falsified^1^. Over the last 20 years, numerous reports describe falsified antimalarials reaching alarmingly high prevalence in hotspots in malarious Asia, Africa and Latin America, driven by problems with access, affordability and corruption^1–8^, as summarized in the Infectious Diseases Data Observatory’s Medicine Quality Scientific Literature Surveyor (https://www.iddo.org/mq-scientific-literature-surveyor)^7^. Falsified medicines result from criminal fraud^1^. They are manufactured in poorly controlled environments and can contain excess active pharmaceutical ingredient (API), or more usually reduced or no API, and toxic adulterants, resulting in preventable mortality and morbidity, unpredictable side effects, increased adverse drug reactions, mistrust in the healthcare system and economic harm. For antimalarial chemotherapy this poses significant threats for malaria control and drug resistance, escalating the scale of this key preventable and treatable disease^9^. It is important to distinguish the criminal enterprise of falsifying medicines from quality deficiencies in legitimate industries which lead to substandard medicines. These result from errors in factories or degradation in impaired supply chains. They are also important impediments to public health but do not involve fraud^1^.

During the COVID-19 pandemic substandard and falsified (SF) medical products, including vaccines, have highlighted our collective vulnerability. This emphasizes the need for innovation to improve our abilities to prevent, detect and respond to this neglected public health issue^1^. However, little research has been conducted to inform detection and forensic strategies for SF medicines^10^. Falsified tablets can be distinguished from genuine products by the physical appearance of the packaging, blisters and tablets (e.g. errors in spelling, poorly copied holograms), physical properties (e.g. disintegration and dissolution), and/or quantification of the API, and excipients present^10^. However, these do not provide objective information on the geographical origin of falsified medicines^10^.

From the late 1990s to the early 2000s there was a large epidemic of falsified oral artesunate across mainland SE Asia. At that time oral artesunate was a widely used treatment for *Plasmodium falciparum* malaria (it has now been replaced by artemisinin-derivative combinations)^6,7^. Falsified artesunate tablets prepared with different recipes were found in neighboring countries, either indicating a common source or a few sources sharing a common distribution network. Falsified tablets of identical physical appearance have been found in diverse countries, suggesting widespread distribution networks extending across multiple borders^3–8^. Therefore exploring the biological signals captured and sealed within the blister pack may provide insight into the local manufacturing environment for public health forensic intelligence to assist with provenance identification.

While pollen and calcite analysis in falsified artesunate tablets indicative of origin led to closure of one trade route and arrests in China and Myanmar^10^, this pollen analysis required a highly skilled palynologist, reference pollen libraries and a time consuming process, which could not identify all pollen/spores to species level. Furthermore, 27% of samples contained no detectable spore/pollen contamination. Environmental DNA (eDNA), genetic material released from organisms into the environment, is increasingly used in forensic investigations^11^. Adventitious contamination of tablets with eDNA (e.g. via direct transfer, airborne dust, aerobiomes) during manufacture and packaging has the potential to provide a distinctive signature associated with tablet provenance. While human DNA has been detected in illicit drug seizures^12^, the use of broader eDNA signatures to understand the origins and trade routes of falsified medicines remains unexplored. Detection and characterization of eDNA within falsified tablets could yield evidence of the origin of falsified medicines and their ingredients; and detection of human DNA could, through ancestry analysis, facilitate prosecution of individuals. Innovation to inform this evidence base at the interface between criminal forensics and public health could have significant health, social and economic impacts in aiding prevention, detection and responding to falsified pharmaceuticals. The introduction of a rapid, accurate and comprehensive environmental DNA screening tool would enable law enforcement agencies to implement and utilize the biological signal within falsified medicines to identify associations between seizures and complement existing tools to build a more complete picture of the trade routes.

Massively Parallel Sequencing (MPS) has revolutionized forensic genetics^13^ and offers the capacity to rapidly generate highly discriminative DNA signals representative of the complex microbial and eukaryotic diversity within samples^13–26^. Microbial signals detected in air^23–25^, water^26^ and negative laboratory controls^27^ highlight that trace environmental DNA (eDNA) characteristic of the surroundings can be detected and could provide a spectral fingerprint characteristic of the ingredients, location and timing of falsified medicine production. This pilot study investigated whether falsified artesunate tablets contain detectable amounts of eDNA and if this signal could be indicative of their production and origin. Tablets in plastic blisters sealed at place of manufacture were used as they would best capture and preserve the diverse genetic signal without further contamination after production. We hypothesized that tablets may contain eDNA integrated into the tablet during production and transit of the ingredients, and transferred to the tablet/inside of the packaging at the source of manufacturing, as well as human DNA from persons involved in preparation (likely transferred by touch and shed skin cells), but also from bystanders. Therefore, we explored the detection of microbial and eukaryote signals using DNA metabarcoding (sample genotype) and whether this signal could potentially be used to differentiate genuine and falsified samples of different packaging types.

## Methods

### Samples

Genuine and falsified samples were confirmed as such by packaging analysis^1,10^. A total of 38 tablets were processed from 11 samples of oral artesunate tablets (Table 1), collected in 2002–2013 from two genuine manufacturers and from pharmacies in Vietnam, Lao PDR (Laos) and on the China/Myanmar border^10^. This included five genuine samples from two suppliers (Guilin Pharmaceutical Co., Ltd and Mekophar Chemical Pharmaceutical Joint Stock Co. Ltd) and six falsified tablet samples (five purchased from a pharmacy in Ho Chi Minh City, Vietnam and one from a pharmacy in Phin District, Savannakhet Province, southern Lao PDR). The five falsified tablet samples purchased in Vietnam had previously been classified based on the packaging type, with two samples as Type 8 and three as Type 11 and the sample from Lao as Type 12). The number of replicate tablets per sample ranged from 1 to 5 for the genuine and 2 to 5 for the falsified tablets. All samples were kept at + 4 °C in the same refrigerators from receipt in Vientiane until dispatch together to the laboratory.

**Table 1:** Sample details: all samples were labelled as containing 50 mg artesunate per tablet. Genuine and falsified samples were confirmed as such by packaging analysis. See (10) for description of packaging types.

Sample code Stated Batch number Manufacture date Expiry date	# Tablets Analysed	Genuine/Falsified & Packaging type and notes	Origin
G015 81004 10/2008 10/2011	5	Genuine. Labelled as made by Guilin Pharmaceutical Co., Ltd	Guilin Pharmaceutical Co., Ltd
Lao 07/04 060107 01/2006 06/2009	4	Genuine. Labelled as made by Guilin Pharmaceutical Co., Ltd	Vientiane, Lao PDR
China 07/10^a^ 050609 06/2005 06/2008	1	Genuine. Labelled as made by Guilin Pharmaceutical Co., Ltd	China/Myanmar border
G218 10005FX 25/03/2010 25/03/2013	2	Genuine. Labelled as made Mekophar Chemical Pharmaceutical Joint Sock Co. Ltd	Mekophar Chemical Pharmaceutical Joint Stock Co. Ltd
G220 10005FX 25/03/2010 25/03/2013	2	Genuine. Labelled as made by Mekophar Chemical Pharmaceutical Joint Sock Co. Ltd	Mekophar Chemical Pharmaceutical Joint Stock Co. Ltd
2/15,058 980201 02/1998 02/2002	5	Falsified. Packaging Type 8	Bought in pharmacy in Ho Chi Minh City, Vietnam
2/15,057 980201 02/1998 02/2002	5	Falsified. Packaging Type 8	Bought in pharmacy in Ho Chi Minh City, Vietnam
2/15,009 10401 04/2001 04/2004	4	Falsified. Packaging Type 11	Bought in pharmacy in Ho Chi Minh City, Vietnam
2/15,011 10401 04/2001 04/2004	4	Falsified. Packaging Type 11	Bought in pharmacy in Ho Chi Minh City, Vietnam
2/15,044 10401 04/2001 04/2004	4	Falsified. Packaging Type 11	Bought in pharmacy in Ho Chi Minh City, Vietnam
Lao 05/17^b^ 040201 02/2004 02/2007	2	Falsified. Packaging Type 12 Dimethylfumerate, starch, calcium carbonate, lactose	Bought in pharmacy, Phin District, Savannakhet Province, southern Lao PDR

^a^pollen analysis^10^ of China 07/10 gave *Pinus* pollen and a few, isolated, large, brown fungal spores, colored (red and blue) and colorless threads with starch.

^b^pollen analysis^10^ of Lao 05/17 gave charcoal, clumped thin-walled cells with outer “perispore”, possibly of algal origin, very few pollen grains – Betulaceae, Poaceae, Sapindaceae pollen and fungal spores.

### DNA extraction

The outer surface of each blister pack was cleaned with 3% sodium hypochlorite and 75% ethanol in a pre-PCR cleanroom. In a dedicated UV hood, individual tablets were removed from the blister pack using sterile tweezers and placed on a fresh aluminum foil sheet; only one tablet was removed from each blister pack at any given time. Using a sterile scalpel blade, ~ 30 mg of tablet material was cut and placed into a 2 ml screw cap tube prefilled with a combination of 1.0 mm and 2.5 mm zirconia/silica beads. A second sample was cut from the same tablet and placed into a separate 2 ml screw cap tube (labelled A and B, respectively). Any remaining tablet material was placed into a third tube and retained for future analysis. Scalpel blades, aluminum foil and gloves were discarded, and the UV hood was cleaned thoroughly, between each tablet sample. Sample material was powdered using two cycles on a Precellys Bead Ruptor at speed 5.0 for 20 s. Following homogenization, two different lysis protocols were applied (*SI Appendix*). For both lysis protocols, the supernatant was processed through the Qiagen DNeasy Mini kit silica spin column following manufacturer’s instructions and eluted in 100 uL of AE buffer. DNA extracts were done in six batches (three per extraction method A1-3, and B1-3) with replicates for each tablet sample spread across different DNA extraction batches to eliminate bias (Table S7). An extraction blank control (EBC) was included in each extraction batch to monitor the background signal.

### Bacterial and eukaryote diversity

For bacterial diversity, the V4 region of the 16S SSU rRNA was targeted using 515F-806R primers^28^, and for eukaryotes, the V9 region of the 18S SSU rRNA was targeted using the Euk1391f-1510rEukBr primers^29,30^. For both targets, the forward and reverse primers were modified to include a 6–8 bp barcode sequence to enable a unique dual barcode combination for each DNA extract and thus minimise risk of contamination associated with laboratory analysis workflow (Table S7). PCR amplifications were performed in triplicate (*SI Appendix*, Methods) and pooled to minimise PCR bias, and a no-template control (NTC) was included for each PCR run to monitor background DNA levels. The purified products were then quantified using the LabChip GX Touch nucleic acid analyzer (PerkinElmer) prior to Adapter Ligation using either the JetSeq Flex Library Kit (Bioline, Meridian Bioscience) or NEBNext Ultra II Library Preparation Kit (New England Biolabs, Ipswich, MA, United States) following the manufacturer’s instructions (*SI Appendix,* Table S7) and pooled into two Illumina MiSeq libraries which were sequenced using a 300 cycle Illumina MiSeq kit at either the Australian Genomics Research Facility (Adelaide) or the South Australian Health and Medical Research Institute (SAHMRI, Adelaide).

Raw reads were demultiplexed based on the P5/P7 index sequences and the unique internal dual barcode combinations and primer sequences were trimmed and analysed in QIIME 2 v 2019.1^31^ (*SI Appendix*, Table S8 and S9; for additional details of the bioinformatic pipeline including taxonomic assignment see *SI Appendix*, Methods). Features with > 100 sequences in the controls (EBCs or NTC) were excluded from the dataset. Alpha diversity (Faith Phylogenetic Distance) and Beta diversity (Bray–Curtis Distance, also expressed as Bray Curtis similarity i.e. 1-dissimilarity) were determined using a rarefaction depth of 1200 and 160 for 16S and 18S, respectively. For 16S, a single extract (Lao_07/04, 5_3B) was excluded, and for 18S, two 15057 extracts (10-2A and 10_3A) and three G015 extracts (14_1B, 14_2B and 14_3A) were excluded due to low sequence count following filtering i.e. less than the respective rarefaction depth. Group significance (genuine/falsified, packaging Type and sample) was calculated using Kruskal–Wallis and PERMANOVA (with 999 permutations) for alpha and beta diversity respectively. PERMANOVA testing is commonly used to test for compositional differences in microbial assemblages, through detection of different group centroids in beta diversity ordination data. In R, the median Bray–Curtis similarity for DNA extracts obtained from a single tablet (termed ‘Within’) and DNA extracts obtained from different tablet samples (termed ‘Between’) was calculated using three sample sets (genuine only, falsified only and all samples combined). Wilcoxon rank sum test and Mann–Whitney test were used to determine the statistical significance between the ‘Within’ and ‘Between’ sample Bray–Curtis similarity for each sample set. This was repeated for both 16S rRNA and 18SrRNA data. Similarly, statistical significance of “Within” sample Bray–Curtis similarity for genuine and falsified tablets was calculated.

For both 16S rRNA and 18S rRNA, differentially abundant ASVs (termed ‘Indicator taxa’) were determined using DESeq2 analysis in the PhyloSeq R package. Indicator taxa were determined for ‘genuine’ vs. ‘falsified’ samples (*SI Appendix*, Figures S4–S5), and within falsified samples for packaging ‘Type 8’ vs. ‘Type 11’ (*SI Appendix*, Figures S6–S7). For display purposes the indicator taxa lists were limited by significance levels (alpha) of 0.0001 and 0.001 respectively. Ternary plots were also developed to explore the relative prevalence of indicator taxa in individual tablet extracts across the falsified packaging Types 8, 11, and 12 (Fig. 4). In this case, packaging type indicator taxa were derived by pooling all significantly increasing and decreasing taxa identified within respective contrasts (Type 11 vs. 8, Type 11 vs. 12, Type 12 vs. 8) in DESeq2 differential abundance testing, at an alpha level of 0.001. The ternary plot performs scaling of the indicator taxa relative abundances so that they sum to unity (100%), and therefore displays their relative composition among the indicator taxa only (not their relative abundance among all taxa).

### Human mitochondrial DNA analysis

Ten falsified tablet DNA extracts were selected for mitochondrial DNA control region analysis, along with a no template control and two extraction blank controls. For each sample, a short, 115 bp fragment of the mtDNA control region spanning positions 16,118–16,232 of the hypervariable region 1 was amplified using primers L16117/H16233^32^ and PCR conditions previously described^33^ (*SI Appendix*, Methods). The hypervariable region 1 is the most polymorphic section of human mtDNA, is the primary target for human forensic mtDNA studies, and has the largest forensic population reference databases available for comparison. PCR products were sent to Australian Genome Research Facility (AGRF, Adelaide, South Australia) for purification and bi-directional Sanger sequencing. Sequence chromatograms were visualized in GeneiousPrime v2020.2.4 (Biomatters) and aligned to the revised Cambridge Reference Sequence (rCRS)^34^. Haplogroup predictions were obtained from the EMPOP mtDNA database (v4/R13)^35–38^ with a restricted range of 16,118–16,232. The frequency of the predicted haplogroups across different geographic populations was determined using the mtDNAmap^39^ and compared to the frequency across different geographic populations described by the 1000 Genomes Project^40^.

To improve the detection of mtDNA and enhance haplogroup and haplotype assignment from the falsified tablets, we also attempted whole mitochondrial genome sequencing on all DNA extracts following the NEBNext Ultra II DNA Library Prep kit as per the manufacturer’s instructions with the exception of custom Y-adapters with internal P5/P7 8 nucleotide barcodes^41^ (at 2.5 nM each) and a half the reaction volume (25 uL DNA extract). Libraries were pooled into two batches (*SI Appendix*, Table S10) and enriched using the Mitochondrial MYTObaits (MYcroarray) following the MYbaits v4.01 protocol (Arbour Biosciences). Enriched libraries were quantified using the 2100 Bioanlayser system (Agilent), pooled and sequenced on one Illumina X ten lane at the Garvan Institute of Medical Research (Sydney, Australia). Raw reads were de-multiplexed by the Illumina software based on the dual P5/P7 index sequences and sequences were de-multiplexed into specific samples based on the dual P5/P7 8-mer internal barcodes in CLC. Fastq files were mapped to the rCRS in GeneiousPrime v2020.2.4 (Biomatters) to determine the percent coverage across the mitogenome. As the sequencing depth was low, haplotype and haplogroup assignment was not conducted.

## Results and discussion

### eDNA profiles

**Figure 1:**
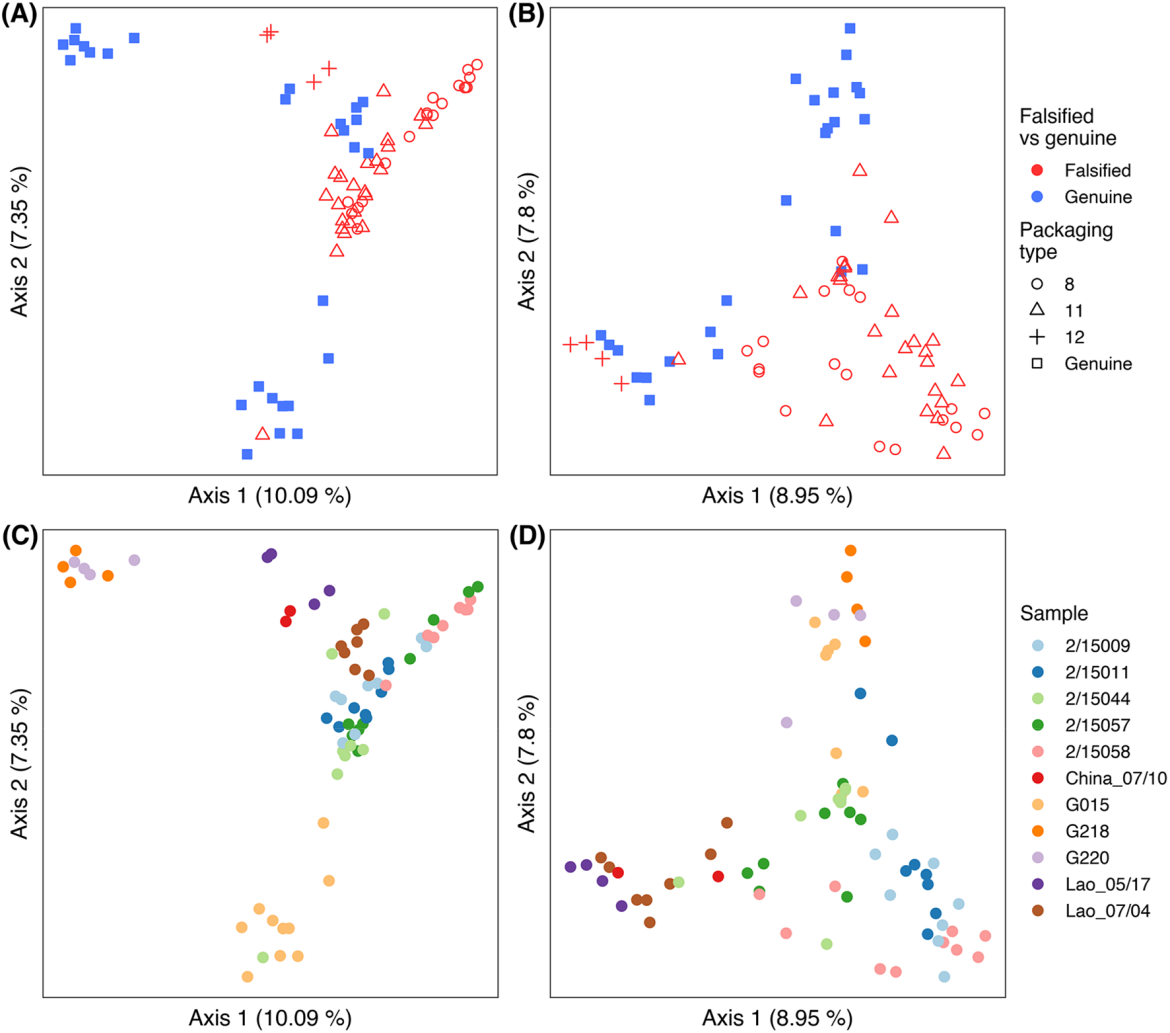
Bray–Curtis Principal Coordinate Analysis of 16S rRNA data in: (**A** and **C**), rarefied at 1200 sequences; and 18S rRNA data in (**B** and **D**), rarefied at 160 sequences. Falsified vs genuine (with packaging type for falsified samples) are shown in (**A** and **B**), while individual samples are shown in (**C** and **D**). PERMANOVA testing (see “Results and discussion ”) reveals compositional differences between genuine and falsified tablets, and between different packaging types (within falsified samples).

This pilot investigation identified the presence of eDNA in both falsified and genuine tablets and provides the first preliminary evidence that such a signal can provide clues to provenance, acquired from the environment in the journey of tablet ingredients into the manufactured product, which are then preserved inside blister packs. We show that eDNA profiles segregate genuine and falsified tablets (Fig. 1A and B, *SI Appendix *Table S1, PERMANOVA: *p* = *0.001* for both 16S and 18S), and for the latter, the different types of falsified samples within a packaging type (Fig. 1C and D, Table S1, PERMANOVA: *p* = *0.001* for both 16S and 18S), regardless of lysis protocol used (*SI Appendix,* Figure S1, *PERMANOVA: 16S, p* = *0.105; 18S p* = *0.999*). Higher bacterial and eukaryote diversity was observed in falsified tablets compared to genuine tablets (Table S1, Faith_PD: 16S, *p* = *0.047; 18S, p* = *0.0003*), which may be attributed to less care and absence of quality control in the unregulated production processes. eDNA profiles observed from individual genuine samples, and individual falsified samples within each packaging type, were also significantly different (Table S1, PERMANOVA: *p* = *0.001* for both 16S and 18S), suggesting that the biotic signals detected are sample-specific (Fig. 1C and D). Pairwise comparisons showed that some samples could only be differentiated using microbial 16S whereas others could only be differentiated using eukaryotic 18S (*SI Appendix, *Table S2 and S3). No significant difference was observed between the following medicines using either marker; China 07/10 and G218, China 07/10 and G220, China 07/10 and Lao 05/17. However, there was only a single replicate for China 07/10 and only two replicates per sample for G220, G218 and Lao 05/17. These genomic data suggest that, as suspected from their appearance and packaging (phenotype), different tablet samples have different origins in space and/or time, and that in their journey from constituent ingredients into blister packs, the tablets have accrued diverse bacterial, fungal, metazoan and plant DNA.

**Figure 2:**
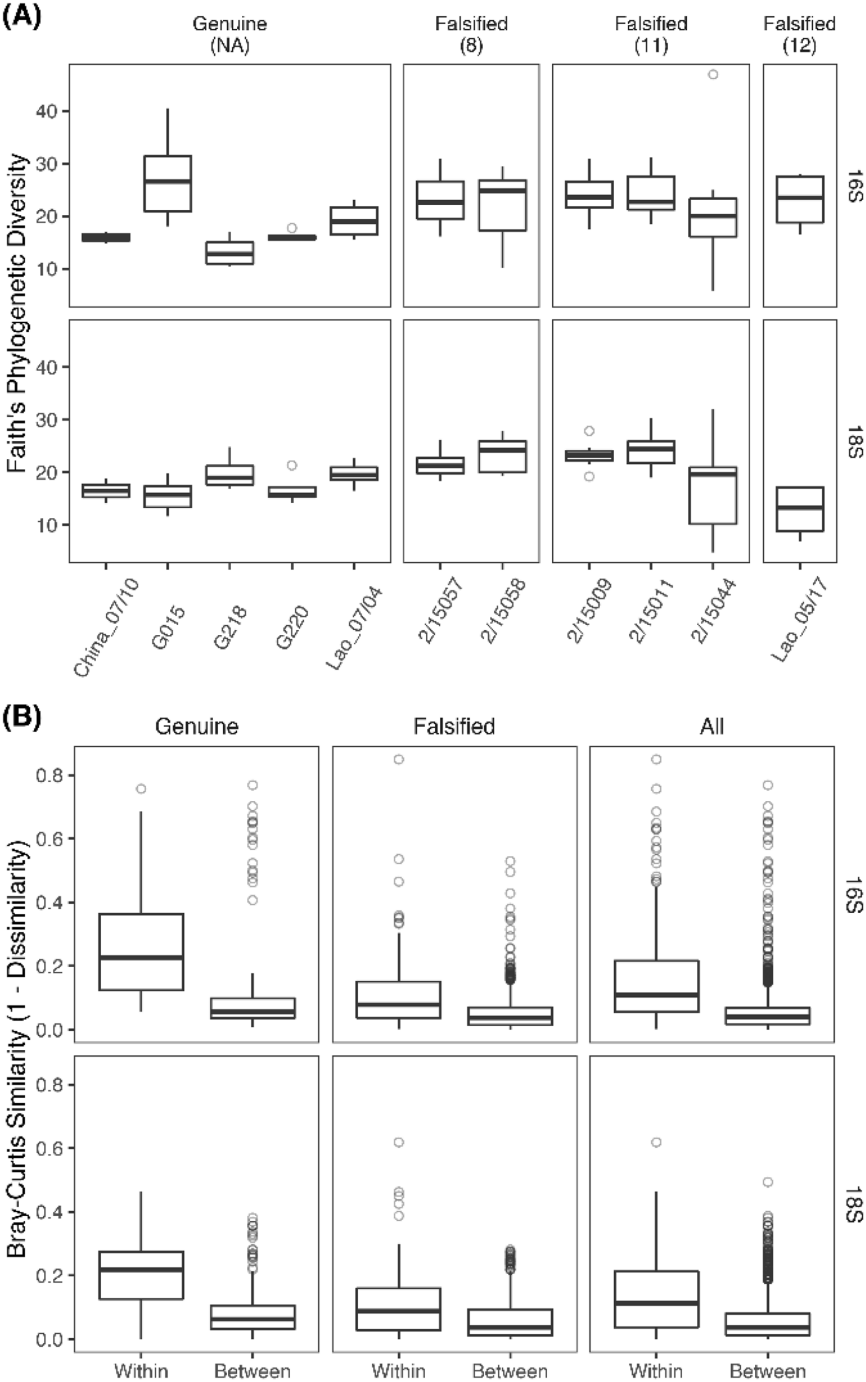
(**A**) 16S and 18S Alpha diversity (Faith PD) classified by tablet type (genuine or falsified) and by packaging type (in brackets) and (**B**) 16S and 18S Bray–Curtis similarities within and between samples for genuine, falsified and all samples.

Whilst genuine and falsified artesunate samples contained different bacterial and eukaryote communities (*SI Appendix*, Figure S2), falsified samples had a significantly higher diversity of eukaryote DNA (*SI Appendix*, Table S1, Faith PD: *p* = *0.0003*) with human DNA also detected (Fig. 5A). Figure 2A displays variation in alpha diversity on a sample-wise basis. The higher detected eukaryote diversity in falsified tablets was supported by a higher number of retained 18S rRNA reads compared to genuine samples (*p* = *0.009*) which was not observed for 16S rRNA (*SI Appendix*, Figure S3, Table S1). Using the Wilcoxon rank sum test/Mann–Whitney significance test, there was a significant difference between ‘Within’ and ‘Between’ sample Bray–Curtis similarity using both 16S (*p* = *2.2e–16)* and 18S rRNA (*p* = *1.229e–10)*, and a higher ‘Within’ sample variability was observed in falsified tablets, compared to the genuine samples (Fig. 2B, and *SI Appendix *Table S4: 16S, *p* = *2.2e–16;* 18S, *p* = *9.294e08*). These results presumably reflect the lack of tightly controlled pharmaceutical manufacturing practices and implementation in the production of falsified tablets, in contrast to the high-quality assurance standards of the genuine pharmaceutical industry.

**Figure 3:**
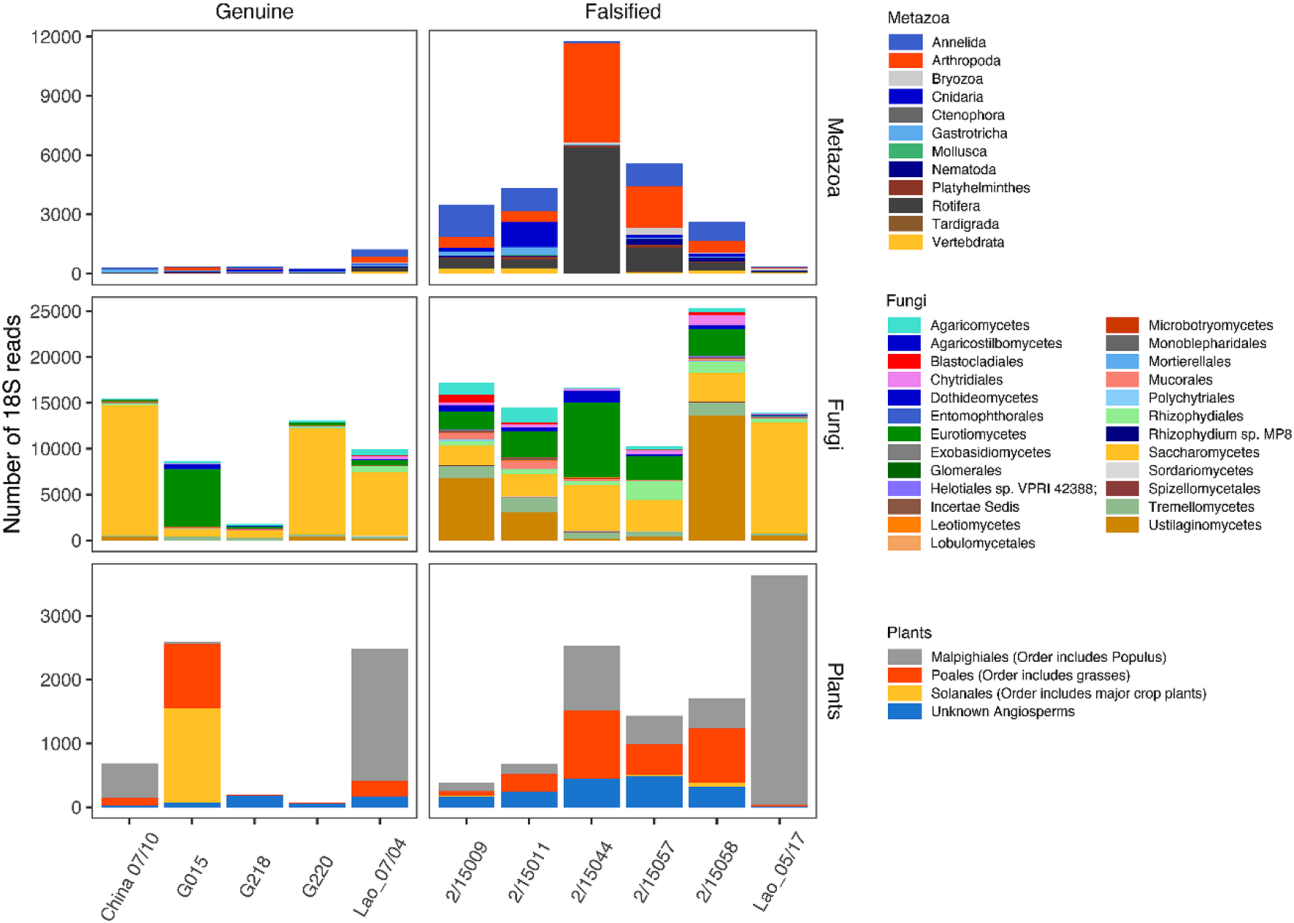
The total number of 18S rRNA sequences assigned to Metazoa (Phylum level), Fungi (Order level) and Plant (Order level) for each genuine and falsified tablet sample. Values represent the total number of sequences detected across all DNA extracts within that sample. * indicates detection of *Zea mays.*

Across all tablet samples fungi represented the highest number of total 18S rRNA sequences per sample, with the exception of G218 (Fig. 3). At order level, Saccharomycetes was dominant in the genuine samples, with the exception of G015 (*SI Appendix,* Figure S2) whereas the falsified samples showed a more even diversity with Ustilaginomycetes, Eurotiomycetes, Tremellomycetes and Rhizophydiales the most dominant, with the exception of Lao 05/17 which was classified as packaging Type 12. Of the Saccharomycetes that generally dominated genuine samples, six species were present in higher abundance in G015 and Lao 07/04 (both from Guilin Pharmaceutical Co. Ltd.) compared to the other three genuine samples, and were not detected in the falsified tablets. Of the 77 16S rRNA ‘Genuine’ indicator taxa (at 0.0001 alpha significance level), all had a relative abundance < 1.5% and only 28.6% could be identified to species. These included *Paenibacillus larvae, Lactobacillus acidiposcis, Aquitalea magnusonii, Cronobacter sakazakii, Massilia timonae, Lactococcus garvieae* and *Streptococcus agalactiae,* whereas ‘falsified’ indicator species (also with a relative abundance < 1.5%) included *Bacillus flexus*, *Pseudomonas azotifigens, Flavobacterium succinicans, Paenibacillus lautus* and *Sphingomonas echinoides* (Table 2, and *SI Appendix*, Figure S4). ‘Genuine’ eukaryote indicator taxa (all with relative abundance < 0.8%) were identified only as the SAR clade (Stramenopila, Alveolata, and Rhizaria) and fungi, with only two identified to species level (*SI Appendix*, Figure S5 and Table S5); *Scheffersomyces coipomoensis*, and *Piskurozyma capsuligena*. Ten ‘falsified’ indicator taxa could be identified to species level (*SI Appendix*, Figure S5 and Table S5). The two species with the highest log2FoldChange associated with a relative abundance > 0.1% in falsified tablets were *Ambomucor seriatoinflatus*, a mucoraceous fungus previously isolated from soil in inner Mongolia and Xinjiang Province, China^42,43^ (detected at highest abundance in packaging Type 11 samples 2/15011 and 2/15009), and *Placus salinus*, a rare marine ciliate previously collected from coastal waters near Qingdao, China^44^. The data presented indicate that while there are differences observed at Order level, rare taxa are driving the discriminatory signals, in agreement with other eDNA studies in forensic science^45,46^, and indicates that the likely origin of tablets/constituents is in east Asia. Limitations of this pilot study include the relatively small sample size and that sample expiry dates were 7–18 years prior to analysis. However, the blisters were all intact and had been kept at + 4 °C since collection in refrigerators. Further work to understand the impact of high temperatures on eDNA profiles in tablets is needed as falsified medicines are traded outside of the regulated supply chains we would expect them to be exposed to higher temperatures than the genuine samples. We are unable to estimate whether the eDNA entered the tablet matrix within the raw ingredients and their sources or during their manufacturing process or both. Further investigation to understand the eDNA spectra in excipients and active pharmaceutical ingredients and ingress during manufacture are needed. Comparison of eDNA within the matrix of tablets, on their surface and in the blister ‘head space’ air may help understand eDNA sources.

**Table 2:** Bacterial species identified as indicator taxa (differentially abundant) for genuine and falsified tablets and for packaging Type 8 or 11. Positive log2FoldChange (G/F) values indicate ‘Genuine’ indicator taxa and negative log2FoldChange (G/F) values indicate ‘Falsified’ indicator taxa. Positive log2FoldChange (8/11) values indicate ‘Falsified Type 8’ indicator taxa and negative log2FoldChange (8/11) values indicate ‘Falsified Type 11’ indicator taxa.

Genuine	log2FoldChange (G/F)	Falsified	log2FoldChange (G/F)
*Paenibacillus larvae*	4.937 (0.208)	Bacillus flexus*	− 6.169
*Lactobacillus acidipiscis*	4.481 (0.077)	Pseudomonas azotifigens*	− 2.910
*Aquitalea magnusonii*^+^	4.359 (1.270)^+^	*Sphingomonas echinoides*	− 2.906
*Cronobacter sakazakii*	4.324	*Flavobacterium succinicans*	− 2.904
*Massilia timonae*	4.264	Paenibacillus lautus*	− 2.314
*Lactococcus garvieae*^+^	4.216 (0.781)^+^		
*Streptococcus agalactiae*	4.072	***Packaging: Type 8***	**log2FoldChange (8/11)**
*Novosphingobium capsulatum*^+^	3.664 (0.885) ^+^	Bacillus flexus*	4.910
*Clostridium acetobutylicum*	3.630	Pseudomonas azotifigens*	3.037
*Acinetobacter rhizosphaerae*	3.341	*Paracoccus marcusii*	2.970
*Enterococcus cecorum*^+^	3.269 (0.571)^+^	*Pedobacter cryoconitis*	2.555
*Aeromonas sharmana*	3.247	Paenibacillus lautus*	2.309
*Lactobacillus coleohominis*	3.107	***Packaging: Type 11***	
*Bifidobacterium animalis*	3.094	*Clostridium perfringens*^*a*^	− 3.827
*Clostridium acetobutylicum*	3.063	*Lactobacillus coleohominis*	− 3.707
*Dyella japonica*	2.832	*Lactobacillus helveticus*^+^	− 3.258
*Lactobacillus delbrueckii*	2.762	*Clostridium perfringens*^*b*^	− 2.951
*Arthrobacter woluwensis*	2.756	*Enterococcus cecorum*^+^	− 2.900
*Lactobacillus helveticus*^+^	2.599 (1.264)^+^	*Brevundimonas diminuta*	− 2.670
*Lactobacillus zeae*	2.573	*Aquitalea magnusonii*^+^	− 2.467
*Selenomonas lacticifex*	2.426	*Novosphingobium capsulatum*^+^	− 2.452
*Clostridium butyricum*	1.686	*Lactococcus garvieae*^+^	− 2.115

*indicates species identified as ‘Falsified’ indicator taxa and ‘Falsified Type 8’ indicator taxa.

^+^indicates species identified as ‘Genuine’ indicator taxa and ‘Falsified Type 11’ indicator taxa. Two ASVs identified as *Clostridium perfringens* (subscript a and b) were differentially abundant in Type 11 samples.

### Sample differences by falsified packaging type

A previous collaborative investigation, that combined chemical, biological and packaging evidence, suggested two different trade routes of falsified artesunate: a westerly group which included samples obtained in Myanmar (Burma), the Thai/Myanmar border and northern Laos; and an easterly group which included samples recovered from southern Laos, Vietnam and Cambodia^10^. Similar pollen signatures suggested that both groups shared a common manufacturing area in southern China, but had different distribution networks. The three falsified packaging types included in this study were from the easterly group only, and shared a common falsified packaging characteristic (the upper-case font error for ‘Mfg’ and ‘Exp’) that was absent from all other easterly samples suggesting a link between their producers^10^. Falsified samples of Type 8 and 11 (purchased in Vietnam) had a higher eukaryote diversity compared to packaging Type 12 samples purchased in southern Lao PDR (Fig. 2A; *SI Appendix *Table S1, *p* = *0.0003*). *Aspergillus cervinus*, a fungi that grows on decaying vegetables, and *Hortaea werneckii*, an extremely halotolerant yeast species, linked to tinea nigra skin infection, common in Asia, Africa and Central America^47^, were detected in all the falsified tablets classified as packaging Types 8 and 11 (*SI Appendix,* Data). *Phellinus noxius OVT-YTM/97*, an invasive brown root pathogen with a pan-tropical/subtropical distribution previously found in Hong Kong^48^, was detected in two falsified samples classified as packaging Type 8 (2/15057) and 11 (2/15009), and in genuine sample China 07/10. *Flammulina velutipes*, enoki or the velvet shank mushroom, which is cultivated on a large scale in east Asia with China currently the largest producer^49,50^, was also detected in a sample from packaging Type 11 (2/15044). Detection of these species supports previously reported distinct Northern Asian temperate pollen and spore signatures (by microscopy) indicative of an origin in eastern Asia centered on southern China and the highlands bordering Myanmar through to Vietnam^10^. The demonstrated capability of eDNA and rapid throughput to discriminate these samples highlights the potential resolution offered by this technique and the probability of identifying distinct signatures between samples originating from different producers. Further eDNA analysis that includes westerly trade route falsified samples would provide further evidence to test this hypothesis and shed new light on the dynamics and interactions of these illegal supply chains.

**Figure 4:**
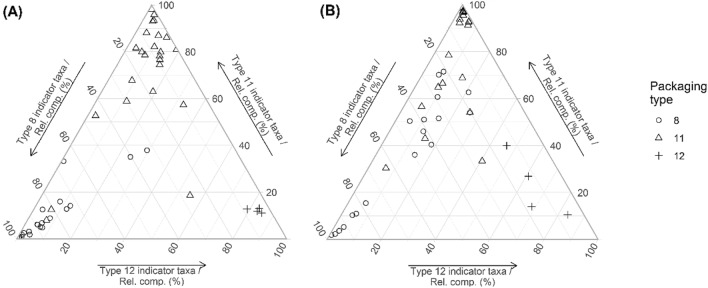
Ternary plots of the percent relative composition of (**A**) bacterial and (**B**) eukaryote indicator taxa for packaging Type 8 (n = 20 tablets), Type 11 (n = 24 tablets), and Type 12 (n = 4 tablets). Indicator taxa were derived by pooling all significantly increasing and decreasing taxa identified within respective contrasts (Type 11 vs. 8, Type 11 vs. 12, Type 12 vs. 8) in DESeq2 differential abundance testing, at an alpha level of 0.001. Note that the % relative composition values displayed here represent scaled data as the relative abundances of indicator taxa for the three packaging types are scaled up to 100% (considering indicator taxa only).

All indicator taxa for packaging Type 8 and 11 (at 0.0001 alpha significance level) had a relative abundance < 1% and < 0.3% for 16S rRNA and 18S rRNA, respectively, with the exception in 16S of *Bacillus flexus*, and two taxa identified only to Order (Bacillales) and genus (*Paenibacillus*) level, which each had a mean relative abundance between 2–4% in packaging Type 8 samples (*SI Appendix,* Figures S6 and S7). Only 25% and 6.6% of 16S and 18S packaging type indicator taxa, respectively, could be identified to species (Table 2, and *SI Appendix,* Table S6). Three diatom taxa were identified as indicators of either packaging type but could only be identified as Bacillariophyceae, while most other indicator taxa belonged to either Fungi, SAR clade or Ciliophora (*SI Appendix,* Table S6). For packaging Type 8 and 12, the relative composition of bacterial indicator taxa detected in the samples was higher than the relative composition of eukaryote indicator taxa (Fig. 4). The term ‘relative composition’ refers to the proportional contribution of a particular set of indicator taxa, among all indicator taxa present (100%). For example, 85% of Type 8 sample extracts had a bacterial indicator taxa relative composition > 70% compared to only 50% of extracts with > 70% eukaryote indicator taxa relative composition. While a similar trend was evident for Type 12 samples, for packaging Type 11 this difference between targets was not observed, only 66% of extracts had a relative indicator taxa composition > 70% for either marker. This suggests that detection of rare discriminating taxa will be sporadic providing an indication of future sampling effort required to capture a representative signal adequately. A more targeted approach would improve the detection of rare indicator taxa, and as this area of research develops, the optimal target genes and taxonomic groups of interest will become more refined.

Previous pollen analysis of falsified artesunate samples detected firs, pines, cypresses, sycamores, alders, wormwood, willows, elms, wattles and numerous fern spores^10^. However, 3/11 (27%) samples contained no detected spore/pollen contamination. In the current study, plant DNA, classified as flowering plants (Angiosperms), was successfully detected in all samples (Fig. 3) indicating the potential to obtain informative genetic plant information from all samples if one, or multiple, gene regions with more genetic variation between plant species were examined^51^. The genus *Populus* (a deciduous tree), and the Order Poales (grasses) were detected in all samples (the former was most prevalent in Lao 05/07 and Lao 07/04) and *Zea mays* (maize) was detected only in falsified samples classified as packaging Type 11 (Fig. 3). Falsified sample 2/15,044 also contained *Triticum aestivum* (common wheat) and *Aegilops tauchii* (Tausch’s goatgrass or rough-spike hard grass), and *Nicotiana* sp. was detected in falsified samples Type 8 and 11 (2/15057, 2/15058 and 15009). *Nicotiana* sp*.* was also detected in genuine sample G015 at a higher level (1109 sequences compared to < 60 sequences for each falsified sample).

### Human DNA

**Figure 5:**
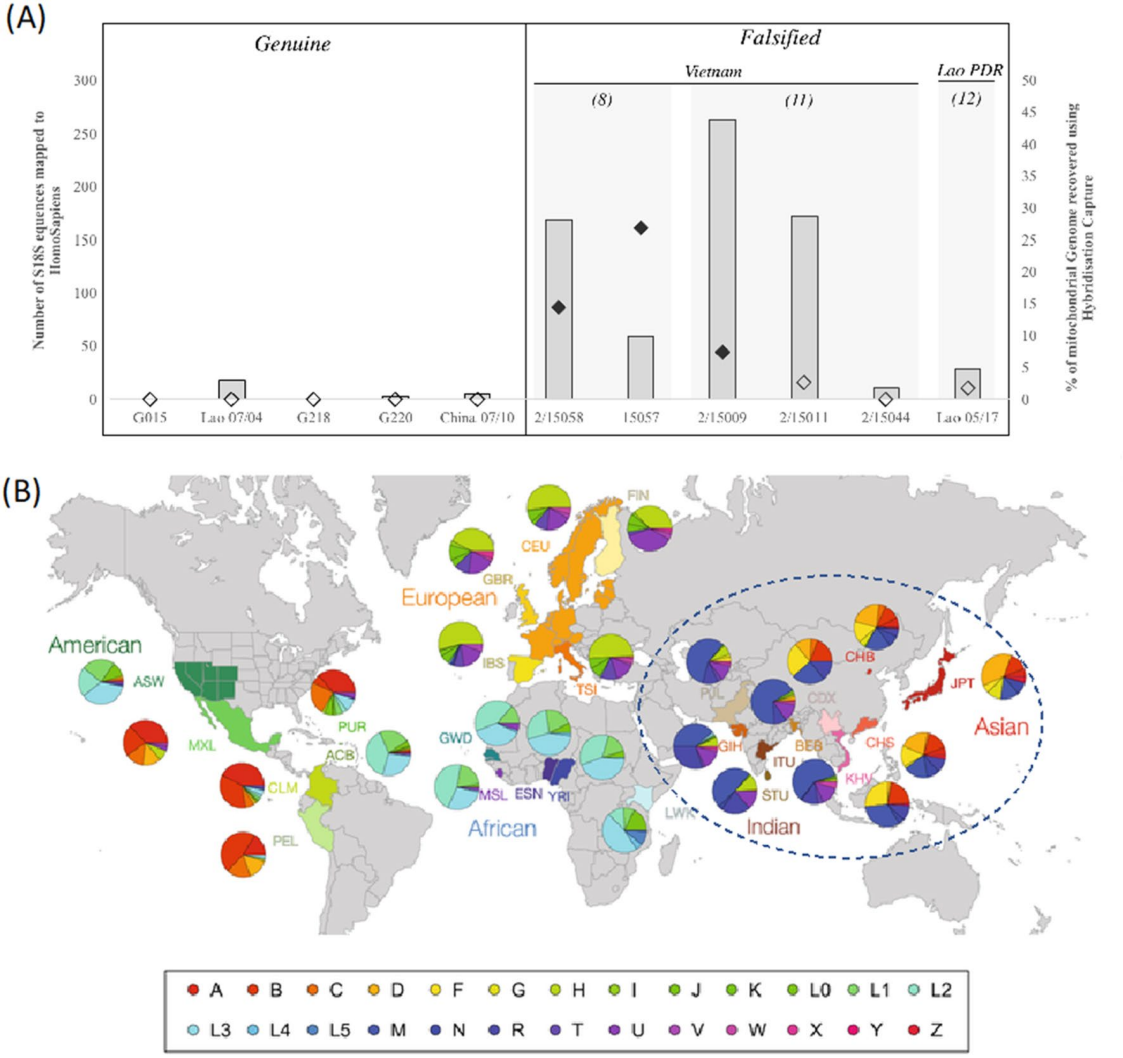
(**A**) Detection of human DNA from tablet extracts using 18S rRNA (as bars) and whole mitochondrial genome hybridization capture analysis (filled diamonds indicate samples where a single source control region sequence (~ 160 bp) was also recovered using PCR). (**B**) The biogeographic ancestry predictions from the mitochondrial control region sequences (blue dotted line) reflected on the frequency of mtDNA subgroups across different geographic populations from the 1000 Genomes Project. The map and haplogroup frequency pie charts have been modified after being reproduced from ^40^ under the Creative Commons Attribution 4.0 International License (https://creativecommons.org/licenses/by/4.0/).

The presence of human DNA in falsified samples raises the prospect of identifying genetic features of individual people whose DNA contaminated the tablets or indeed the identity of those manufacturing them legally or illegally and of uninvolved bystanders. Falsified samples classified as packaging Type 8 and 11 showed the highest total number of *H. sapiens* 18S sequences (Fig. 5A) with detection in 70% Type 8 samples (5/5, 2/15058; 2/5, 2/15057) and in 67% Type 11 samples (4/4, 2/15009; 3/4, 2/15011; and 1/4, 2/15044). In contrast, falsified tablet Type 12, and the genuine samples had < 30 *H. sapiens* sequences. Five falsified tablet DNA extracts with the highest 18S reads classified as *H. sapiens* also produced good quality, single source mitochondrial control region sequences suggesting a single donor; these were also the DNA extracts that yielded the most successful mitochondrial capture results (Fig. 5A). Mitochondrial control region sequences from 2/15057 (Type 8) and 2/15009 (Type 11) indicate DNA from an individual(s) belonging to haplogroup M, D or E (*SI Appendix,* Figure S8A). Both haplogroup M and D are present at a much higher frequency in Asian populations compared to other global populations; haplogroup M predominates in west Asia whereas haplogroup D predominates in East Asia (Fig. 5B). Haplogroup E is typical for the Malay Archipelago and the suggested subgroup E1a1b3 (as predicted by EMPOP) is present in the Filipino population (*SI Appendix,* Figure S8B). Two of the suggested subgroups of M are present in the Chinese population. For M7c1c2, the highest frequency is in Hong Kong although it is also present in Yunnan (China), Vietnam, Thailand, and Laos (*SI Appendix,* Figure S8B). Similarly, M7c1a3 is also present in the Yunan population despite higher frequency in west Asia (Afghanistan, India and Buryat Republic). The potential east Asian ancestry is also supported by sequences recovered from 2/15057-4A and 15058-4B, where the potential subgroup of D (D4c1b) is present in China, Mongolia, Afghanistan and Uzbekistan. In this study, trace levels of human DNA were detected within the tablet matrix. However, the possibility of detecting human DNA from the packaging using a fluorescent dye to visualize cellular material^52,53^ coupled with direct PCR methods^54^, as well as other ancestry informative markers^55,56^, could also be applied. Detection of such DNA could represent people actively involved in criminal activities as well as those innocent of such fraud, whose DNA was transferred in and on the constituents or were near the site of production. Therefore, this will raise many practical, legal and ethical issues, risks and benefits, that need further consideration as these methods are developed.

## Conclusion

With further research it should be possible to build eDNA profiles of tablets that reflect their journey into the blisters. The techniques could also be used for medicines in capsules and medicines and vaccines in vials/syringes. One key issue that confounds the results is that the genomic assemblages will reflect the aggregate eDNA of the tablet components. For example, artesunate is derived from *Artemisia annua* and tablets may include maize flour, both plant products. So the artesunate tablets may include eDNA from the plant ingredients, from the fields in which they were grown, the container in which it was transported, the air and water in the factory where the artesunate was manufactured, the plastic used to make the blisters and the people, whether involved or bystanders, who came close to the ingredients. Hence, each encapsulated tablet will contain a ‘pharmabiome’ representing the ingress of eDNA at different stages in the creation of that tablet and should be characteristic of those processes in those environments and at that time. As such, we would expect the pharmabiome to vary through time and space as the surrounding eDNA envelope varies. Despite the enormous global public health importance of medicine quality, there has been very limited research and funding to address this final common pathway to what patients consume. The ability of data from this novel technique to inform manufacturing origins will be dependent on the growing availability of accurate databases of the spectra of eDNA globally and how these vary through time and space. Further work is needed using the creation of simulated medicines in different environments to understand the accrual of eDNA at different stages of the creation of tablets and how these spectra differ between different environments and seasons.

## Supplementary Information

Supplementary Information.

## Data Availability

The datasets generated and analysed during the current study are available in the Sequence Read Archive (accession number PRJNA860729).
