# Supplementary material for: Environmental DNA as an innovative technique to identify the origins of falsified antimalarial tablets—a pilot study of the pharmabiome
Source: Sci Rep. 2022 Dec 20;12:21997. doi: 10.1038/s41598-022-25196-0 (PMC9764312; doi:10.1038/s41598-022-25196-0)
Supplement: Supplementary file 1 — Supplementary Information. [file 41598_2022_25196_MOESM1_ESM.pdf]

Supplementary Information for

Environmental DNA as an innovative technique to identify the origins of falsified antimalarial tablets - a pilot study of the pharmabiome

Jennifer M. Young[1*], Craig Liddicoat[1,2], Kor-jent van Dijk[3], Patricia Tabernero[4-7], Celine Caillet[4-6], Nicholas J. White [6,8] Adrian Linacre[1], Jeremy J. Austin[9], Paul N. Newton[4,5,6,8,10,11*]

[1]College of Science and Engineering, Flinders University, Adelaide, South Australia 5042, Australia

[2] School of Public Health, The University of Adelaide, Adelaide, South Australia 5005, Australia

[3]Advanced DNA, Identification and Forensic Facility, School of Biological Sciences, University of Adelaide, South Australia 5005, Australia

[4] Lao-Oxford-Mahosot-Hospital-Wellcome Trust Research Unit (LOMWRU), Microbiology Laboratory, Mahosot Hospital, Vientiane, Lao PDR

[5] Centre for Tropical Medicine and Global Health, Nuffield Department of Medicine, University of Oxford, OX3 7LJ, UK

[6]Infectious Diseases Data Observatory, Nuffield Department of Medicine, University of Oxford, OX3 7LJ, UK

[7] Public Health Unit, Faculty of Medicine, University of Alcalá, 28871 Alcalá de Henares, Spain

[8] Mahidol Oxford Research Unit, Faculty of Tropical Medicine, Mahidol University, Bangkok, 10400, Thailand

[9]Australian Centre for Ancient DNA, School of Biological Sciences, University of Adelaide, Adelaide, South Australia 5005, Australia

[10] Faculty of Infectious and Tropical Diseases, London School of Hygiene and Tropical Medicine, London, WC1E 7HT, UK

[11] Boston University School of Public Health, Boston, MA 02118, USA

* Jennifer M. Young, Paul N. Newton

Email: Jennifer.young@adelaide.edu.au, Paul.newton@tropmedres.ac;

This pdf includes: Methods
References (SI Appendix Text)
Figures S1- S8
Tables S1- S10

Methods.

DNA Extraction

Lysis Protocol A: 945 uL of 0.5M EDTA (pH 8), 20 uL of 10% SDS and 20 uL of 20mg/ml Proteinase K was added to the homogenized powder and agitated at 56°C for 24 hours at 1000 rpm (Eppendorf Thermomixer). Samples were centrifuged at 16,000xg for 5 minutes to pellet any remaining powder. The supernatant was removed and 985 uL AL buffer (from the Qiagen DNeasy Mini kit) was added and mixed at room temperature for 10 minutes at 700 rpm (Thermomixer). Using this lysis protocol, some samples produced a white paste after addition of 100% ethanol (likely due to the presence of calcium carbonate) which required an additional centrifugation step prior to the spin column.

Lysis Protocol B: 900 uL of lysis buffer (from a timber DNA extraction method (1)), 45 uL of DTT (1M) and 18 uL of Proteinase K (20 mM/mL) were added to the homogenized powder and agitated on the thermomixer for 5 hours at 55°C at 600 rpm and then centrifuged at 16,000xg for 5 minutes to pellet any remaining powder. The supernatant was transferred to a 5 mL tube and 960 uL AL buffer (or equivalent volume was added) and mixed at room temperature for 10 minutes at 700 rpm (Thermomixer).

Bacterial and Eukaryote Diversity

<u>PCR Amplification.</u>
All PCR amplifications were performed in a 12.5 µL reaction mix containing 0.3µM of each primer and 1.25 µL DNA extract in KAPA HiFi HotStart ReadyMix diluted to 1x (Kapa Biosystems, Inc., Wilmington, MA, USA). The PCR amplification protocol was 3 mins at 95°C, followed by 35 cycles of 95°C for 20s, 55°C 15s, and 72°C for 30 s, and a final extension at 72°C for 10 mins. PCR amplifications were performed in triplicate and pooled to minimise PCR bias, and a no-template control (NTC) was included for each PCR run to monitor background DNA levels. The pooled triplicate PCR products were quantified using a LabChip GX Touch Nucleic Acid Analyzer (PerkinElmer, Waltham, MA, United States) and were pooled into groups of approximately 20 samples before being purified using the Agencourt AMPureXP PCR Purification process (Beckman Coulter Genomics, Chaska, Minnesota, United States). The purified products were then quantified using the LabChip prior to Adapter Ligation.

<u>Adapter Ligation.</u>
Sequencing Run 1: Three individual tablets from three samples (G015, 15057 and 2/15044) were sequenced primarily (n=18 plus EBCs and NTC, Table S1). In this instance, PCR set up was done manually in a dedicated UV hood and Illumina TruSeq adapters were added using the Bioline JetSeq Flex Library Kit following the manufacturer's instructions. Purified PCR products were pooled into four batches (2 x16S, 2 x18S) representing ~12 DNA extracts in each. The resulting 16S PCR pools were diluted to 1 ng/uL and 18S PCR pools were diluted to 0.5 ng/uL. An adapter concentration of 4 uM was used in the ligation reaction and a unique P7 index was incorporated to differentiate the two markers during analysis: a universal P5 adapter was used. Following adapter ligation and purification using Agencourt AMPure XP at 0.8X (Beckman Coulter Genomics, NSW), all pools were quantified using an Agilent 2200 TapeStation (Agilent Technologies, Santa Clara, CA, USA) and combined at equimolar concentration. The final library was diluted to 4 nM and sequenced using a 300 cycle Illumina MiSeq kit at the Australian Genomics Research Facility (Adelaide).

Sequencing Run 2: For the remaining DNA extracts (n=58 plus EBCs, Table S1), PCR amplification was done on an Eppendorf Epmotion Robot, and the Illumina TruSeq Adapters were added using the NEBNext Ultra II Library Preparation Kit (New England Biolabs, Ipswich, MA, United States) following the manufacturer's instructions. Purified PCR products were pooled into six batches (3 x 16S, 3 x 18S), each representing either 20 or 24 DNA extracts, and a unique P5 and P7 index combination was incorporated for each sample (Table S1) with a working adapter concentration of 15uM. DNA input for 16S pools and 18S pools ranged from 6-10 ng/uL and 16-36 ng/uL respectively. Following adapter ligation and purification using Magna Beads (2), all

pools were quantified using an Agilent 2200 TapeStation (Agilent Technologies, Santa Clara, CA, USA) and combined at equimolar concentration. The final library was diluted to 4 nM and sequenced using a 300 cycle Illumina MiSeq kit at South Australian Health and Medical Research Institute (SAHMRI, Adelaide).

Data Analysis.

Raw reads were demultiplexed into the adapter ligation pools using the P5/P7 index sequences on the Illumina software. In CLC Main Workbench (QIAGEN v12), fastq files were demultiplexed into DNA extracts using the unique dual barcode combinations and primer sequences were trimmed using default settings in 'trim sequences' function. All trimmed fastq files from Run 1 and Run 2 were exported from CLC and imported into QIIME 2 v 2019.1 (3) in 'PairedEndFastqManifestPhred3' format: files for 16S rRNA and 18S rRNA were imported separately. In QIIME 2, sequences were quality filtered using deblur and dada2 (both with trim length of 120 bp) for 16S and 18S, respectively. For both datasets, features with >100 sequences in the controls (EBCs or NTC) were excluded from the sample data. Linear Regression in R was used to determine statistical difference in the number of retained reads for genuine/falsified, packaging type and sample (Table S4).16S rRNA taxonomy was assigned using the Greengenes 99% OTU sequence and taxonomy reference data (trunc-len 120, min length 100, max length 400) and a Naïve Bayes classifier, and 18S rRNA taxonomy was assigned using the Silva_128_99_classifier. Alpha diversity (Faith Phylogenetic Distance) and Beta diversity (Bray-Curtis Distance) were determined using a rarefaction depth of 1200 and 160 for 16S and 18S, respectively.

Human Mitochondrial Control Region Analysis.

Based on detection of *H. sapiens* in the 18S rRNA analysis, 10 falsified tablet DNA extracts were selected for mitochondrial DNA control region analysis. For 2/15058, 2/15009 and 2/15058, two DNA extracts originating from two separate tablets were analysed and for sample 2/15057, both DNA extracts from two tablets were analysed. For each sample, a short, 115 bp fragment of the mtDNA control region spanning positions 16,118–16,232 of the hypervariable region 1 was amplified using primers L16117/H16233 and PCR conditions previously described (4, 5). The hypervariable region 1 is the most polymorphic section of human mtDNA, is the primary target for human forensic mtDNA studies, and has the largest forensic population reference databases available for comparison. The selected amplicon is the shortest (of four overlapping amplicons) and most reliable (in terms of amplification and sequencing success) that has been routinely used for human hypervariable region I sequencing (e.g. 4, 5). PCRs were done in 25 uL volumes containing 1x High Fidelity Buffer (Thermo Fisher Scientific), 1 mg/mL Rabbit Serum Albumin (Sigma), 2 mM $MgSO_4$, 250 uM each dNTP, 0.5U Platinum Taq High Fidelity (Thermo Fisher Scientific), 400 nM forward primer, 400 nM reverse primer, and 2 uL of DNA. Each primer included an M13 tag to enable sequencing of all amplicons with the same sequencing primers. Thermocycling conditions were 94°C for 2min followed by 50 cycles of 94°C for 15s, 55°C for 15s, and 68°C for 30s, followed by 10min. at 68°C. A no template control and two extraction blank controls were included. PCR products were visualized via electrophoresis on a 3.5% agarose TBE gel and those from the tablet DNA extracts were sent to Australian Genome Research Facility (AGRF, Adelaide, South Australia) for purification and bi-directional Sanger sequencing. Sequence chromatograms were visualized in GeneiousPrime v2020.2.4 (Biomatters) and aligned to the revised Cambridge Reference Sequence (rCRS) (6). Haplogroup predictions were obtained from the EMPOP mtDNA database (v4/R13)(7- 10) by submitting haplotypes with a restricted range of 16118-16,222. The frequency of the predicted haplogroups across different geographic populations was determined using the mtDNAmap (11) and compared to the frequency across different geographic populations described by the 1000 Genomes Project (12).

References

1. A. Lowe, et al. A method of extracting plant nucleic acids from lignified plant tissue. International Patent Number WO/2015/070279 (2015).
2. N. Rohland, D. Reich. Cost-effective, high-throughput DNA sequencing libraries for multiplexed target capture. Genome Res. 22 5:939-46(2012).
3. E. Bolyen, et al. Reproducible, interactive, scalable and extensible microbiome data science using QIIME 2. Nat. Biotechnol. 37 8:852-57(2019).
4. J.M. Young, D. Higgins, J. Austin. Hybridization enrichment to improve forensic mitochondrial DNA analysis of highly degraded human remains. Front. Ecol. Evol. 7:450(2019).
5. W. Haak, et al. Ancient DNA from the first European farmers in 7500-year-old Neolithic sites. Science 310 5750:1016-18(2005).
6. R.M. Andrews, et al. Reanalysis and revision of the Cambridge reference sequence for human mitochondrial DNA. Nat. Genet. 23 2:147-47(1999).
7. W. Parson, A. Dür. EMPOP—a forensic mtDNA database. Forensic Sci. Int. Genet. 1 2:88-92(2007).
8. W. Parson, et al. DNA Commission of the International Society for Forensic Genetics: revised and extended guidelines for mitochondrial DNA typing. Forensic Sci. Int. Genet. 13:134-42(2014).
9. B. Zimmermann, et al. Application of a west Eurasian-specific filter for quasi-median network analysis: sharpening the blade for mtDNA error detection. Forensic Sci. Int. Genet. 5 2:133-37(2011).
10. N. Huber, W. Parson, A. Dür. Next generation database search algorithm for forensic mitogenome analyses. Forensic Sci. Int. Genet. 37:204-14(2018).
11. H. ur Rasheed, M. Jawad, S. Nazir, S. Noreen, A. Rakha. mtDNAmap: Geographic representation of mtDNA Haplogroups. Forensic Sci. Int. Genet. Suppl. Ser. 6:e516-e17(2017).
12. L. Rishishwar, I.K. Jordan. Implications of human evolution and admixture for mitochondrial replacement therapy. BMC Genomics. 18 1:1-11(2017).

Supplementary Figures

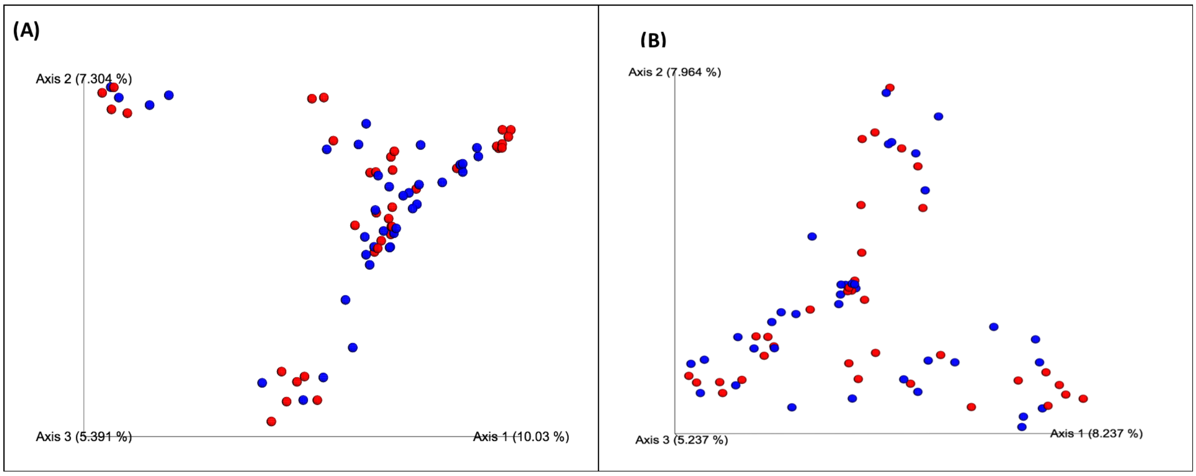

Figure S1: Bray-Curtis Principal Coordinate Analysis of (A) 16S rRNA (rarefied at 1200 sequences) and (B) 18S rRNA (rarefied at 100 sequences) grouped by Lysis Protocol A (Red) and Protocol B (Blue). PERMANOVA: 16S, p=0.105, t-statistic = 1.255; 18S p=0.999, t-stat=0.648.

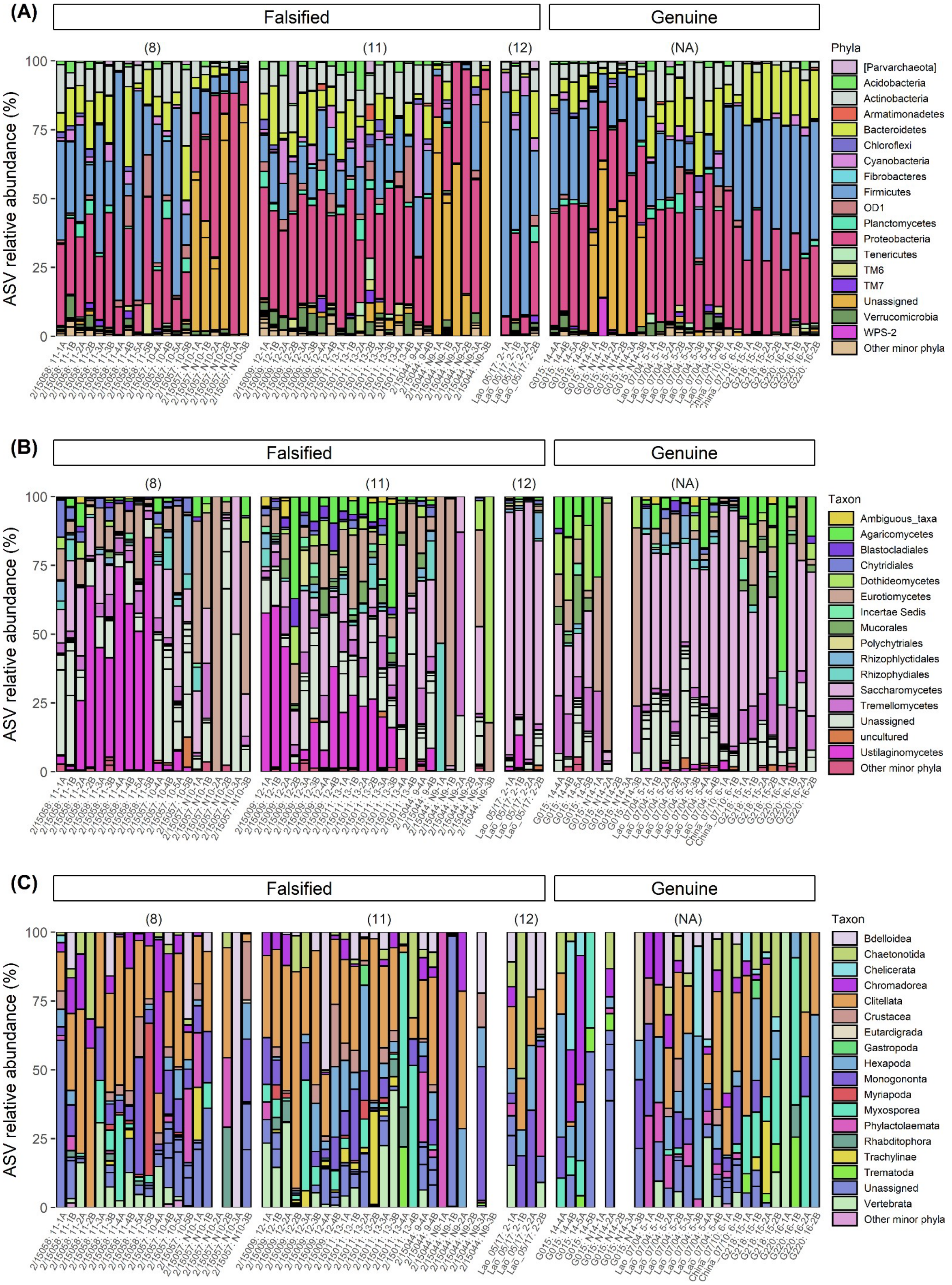

Figure S2: Amplicon sequence variant (ASV) relative abundance bar plots for (A) Bacteria, (B) Fungi, (C) Metazoa, grouped by falsified vs. genuine, and packaging type (in brackets). Missing columns indicate absence of the particular taxon in those samples. Common starting numbers in the sample identifiers listed along the bottom indicate common samples.

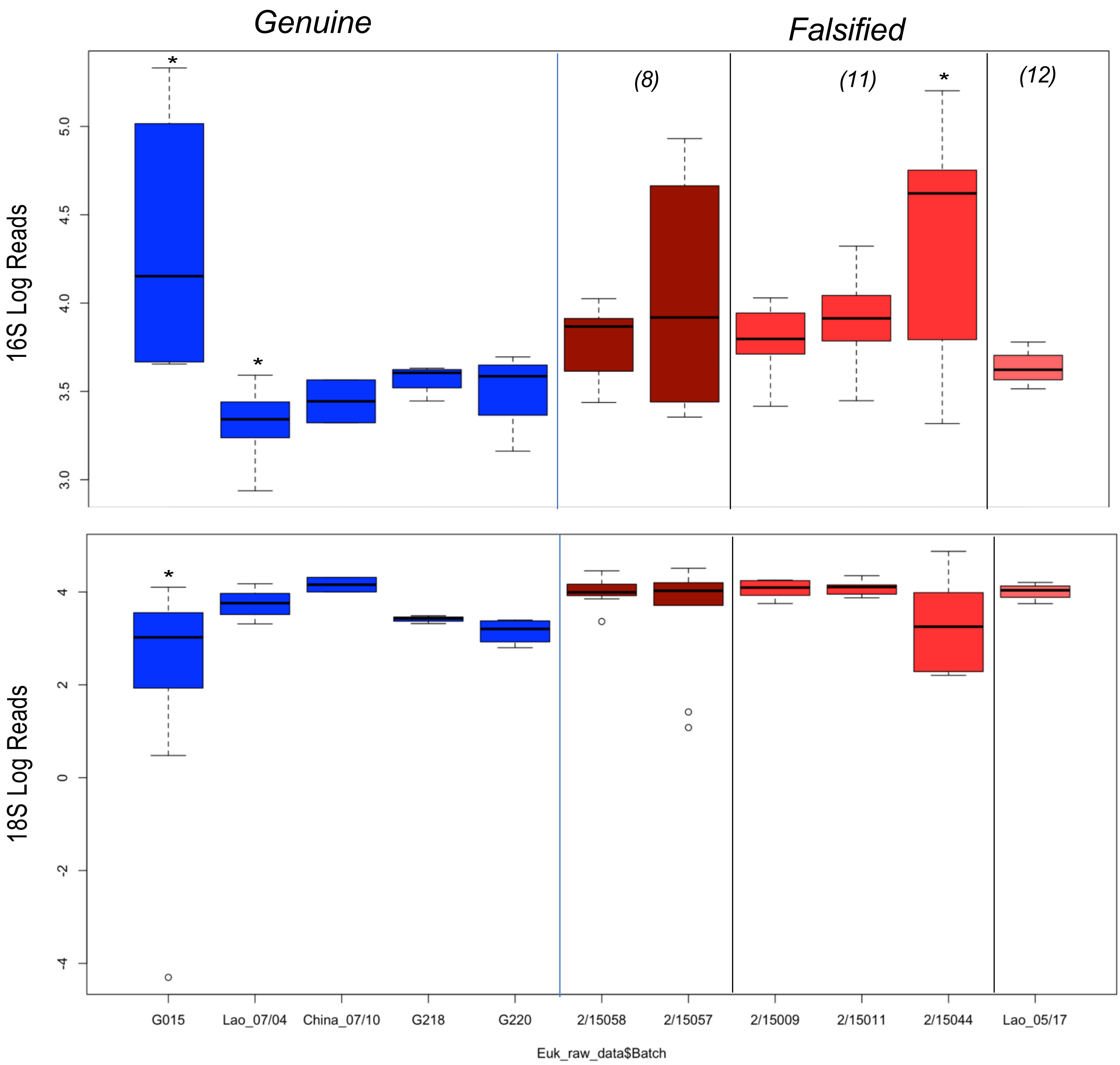

Figure S3: Number of reads retained in the genuine and falsified tablet samples after quality filtering and removal of sequences detected in control sample.

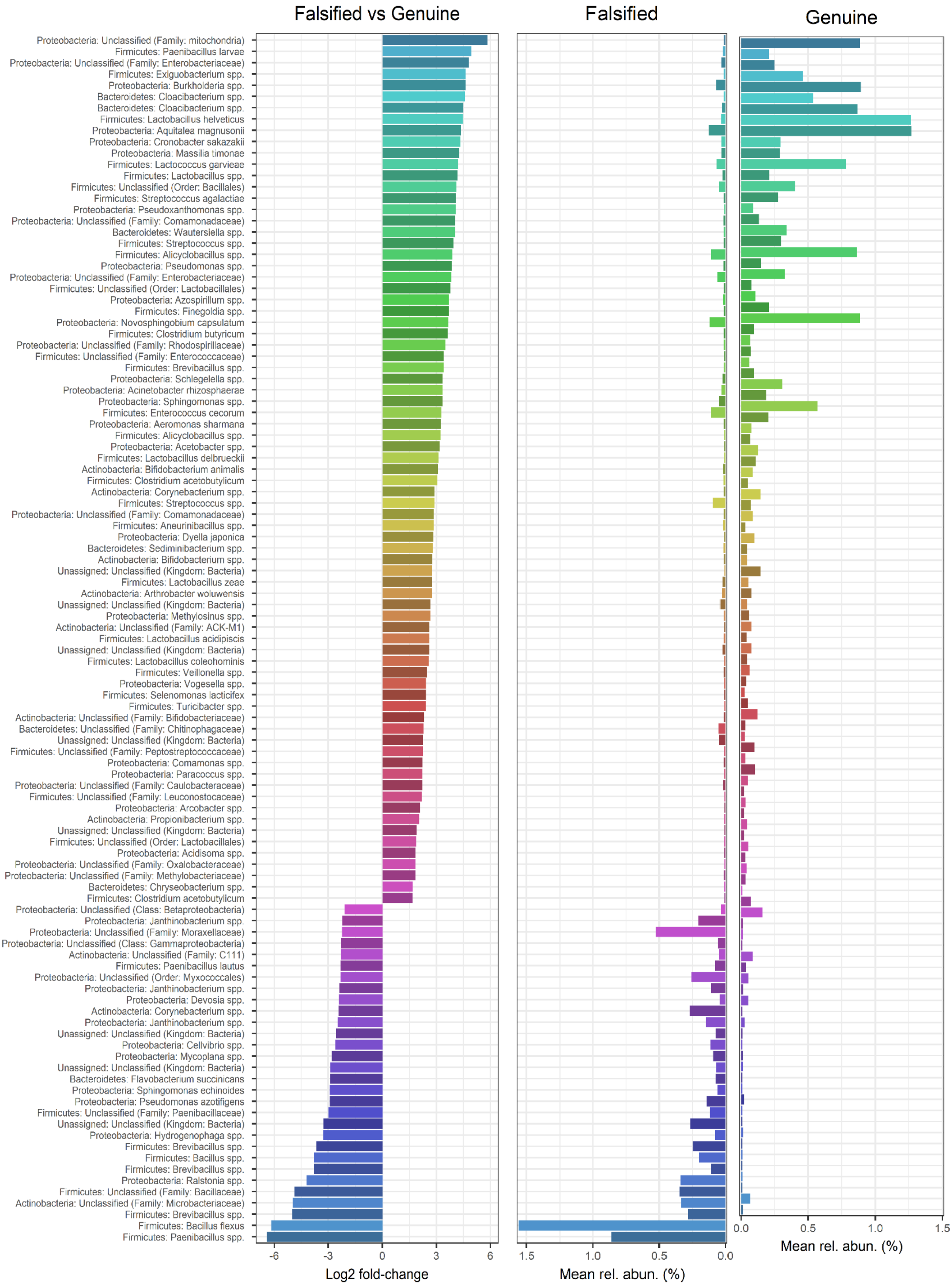

Figure S4: Differentially abundant bacterial ASVs between falsified (n = 48) and genuine (n = 28) tablets. The panels show the log2 fold-change between falsified and genuine tablets (at a significance level of alpha = 0.0001), and also the mean relative abundance (%) of taxa within the respective groups. Taxa (n = 107) are annotated with Phylum, Genus and Species where available, or otherwise to the nearest available classification.

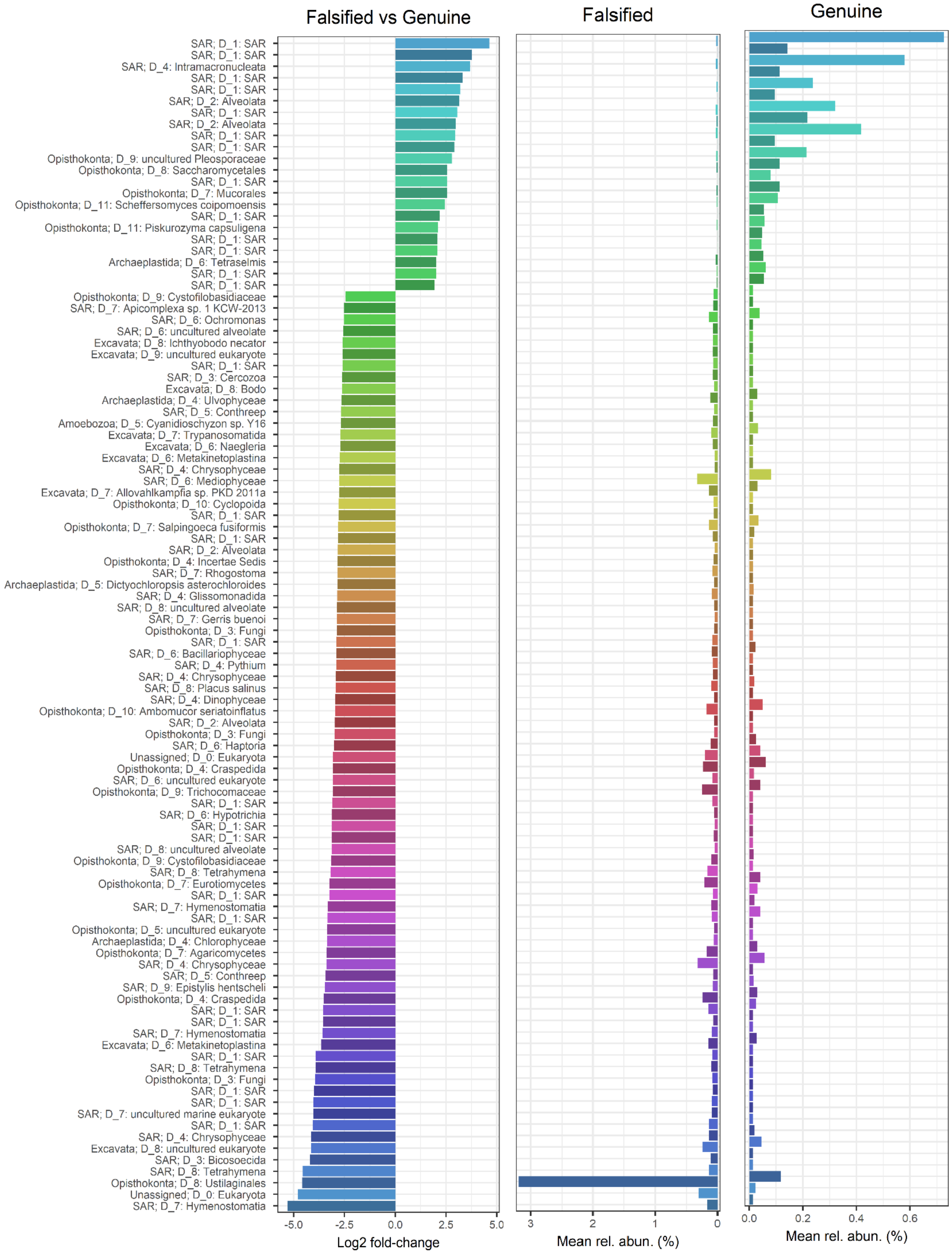

Figure S5. Differentially abundant eukaryote ASVs between falsified (n = 48) and genuine (n = 27) tablets. The panels show the log2 fold-change between falsified and genuine tablets (at a significance level of alpha = 0.00001), and also the mean relative abundance (%) of taxa within the respective groups. Taxa (n = 102) are annotated at the D_1 taxonomy level and also to the finest available classification. Note the different scales for mean relative abundance.

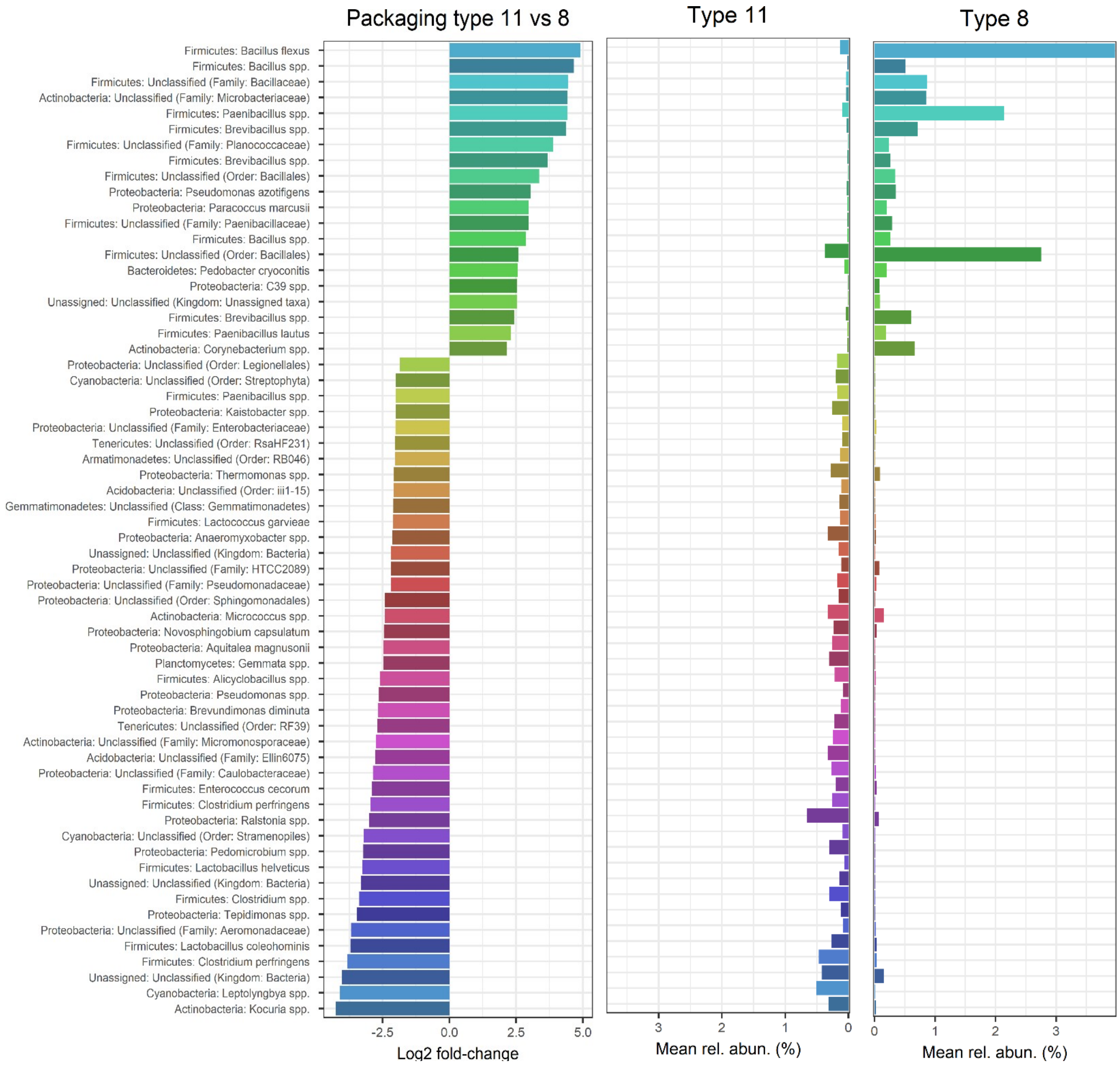

Figure S6. Differentially abundant bacterial ASVs in Falsified-only tablets, between packaging Type 11 (n = 24) and packaging Type 8 (n = 20). The panels show the log2 fold-change between packaging Type 11 and packaging Type 8 tablets (at a significance level of alpha = 0.001), and also the mean relative abundance (%) of taxa with the respective groups. Taxa (n = 62) are annotated with Phylum, Genus and Species where available, or otherwise to the nearest available classification.

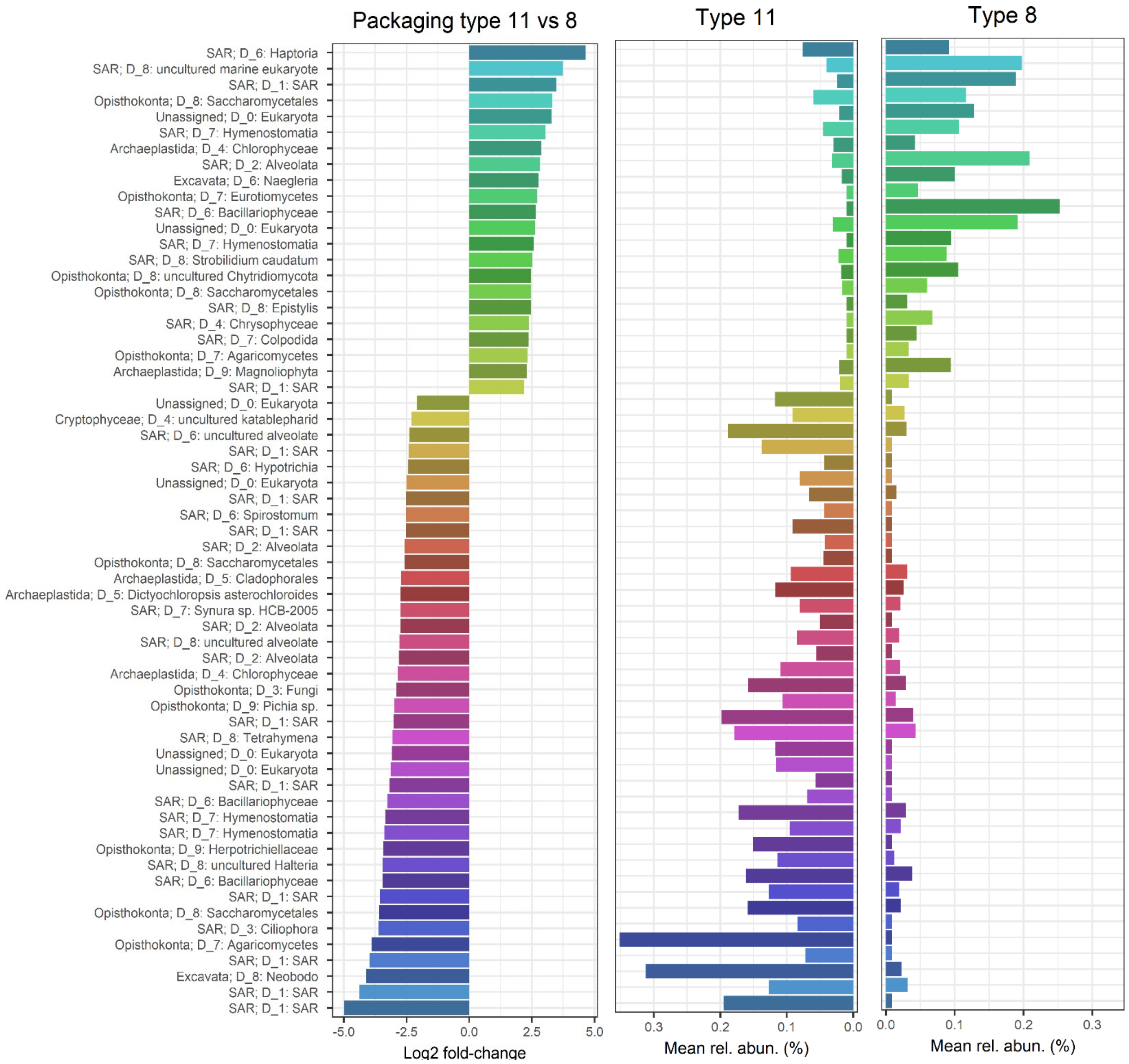

Figure S7: Differentially abundant eukaryote ASVs in falsified tablets, between packaging Type 11 (n = 24) and packaging Type 8 (n = 20). The panels show the log2 fold-change between packaging Type 11 and packaging Type 8 tablets (at a significance level of alpha = 0.001), and also the mean relative abundance (%) of taxa with the respective groups. Taxa (n = 61) are annotated with Phylum, Genus and Species where available, or otherwise to the nearest available classification.

(A)

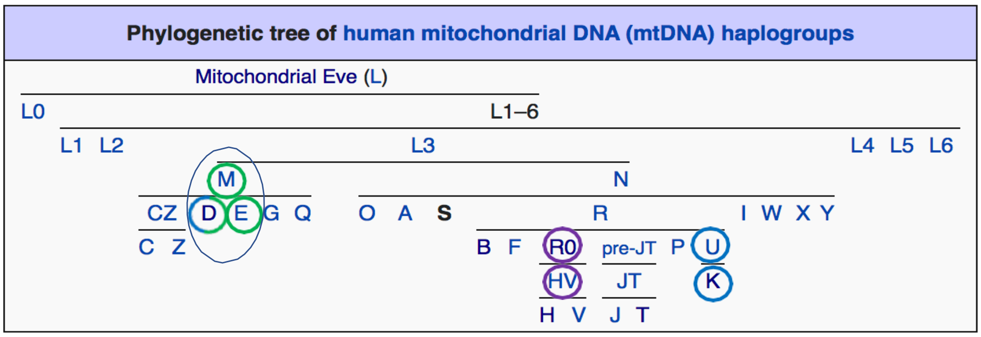

(B)

Haplogroups	Country (Population)	Frequency
M7c1c2	**China (Hong Kong)**	**0.016**
	**China (Yunnan)**	**0.005**
	Vietnam (Kin)	0.014
	Vietnam (Vietnamese)	0.005
	Thailand	0.005
	Laos	0.005
M7c1a3	Afghanistan	0.008
	Buryat Republic	0.005
	India	0.011
	**China (Yunnan)**	**0.001**
M52a1b1	Iran	0.033
E1a1b3	**Philipine**	**0.002**
	Kemerova (Russia)	0.036
D4c1b	**Mongolia**	**0.021**
	Afghanistan	0.007
	**China (Han)**	**0.006**
	Uzbakistan	0.003

Figure S8: The human mtDNA biogeographic ancestry predictions detected using control region sequence recovered from tablet DNA extracts. (A) The predicted mitochondrial haplogroup of three different control region sequences detected across five DNA extracts projected onto the phylogenetic tree of mtDNA haplogroups. (B) The frequency of predicted mtDNA sub-haplogroups (represented in more than one DNA extract) across different geographic populations.

Colours indicate groups of tablet DNA extracts with identical CR sequence: Green= 2/15057-5A and 2/15009-1B, Blue = 2/15057-4A and 2/15058-4B, Purple = 2/15057-4B.

Supplementary Tables

Table S1: Statistical analysis of Beta diversity (PERMANOVA), alpha diversity (Faith_FD) and the number of reads retained after quality filtering (linear regression) for 16S rRNA and 18S rRNA for three comparisons. Bold values indicate statistical significance (*p<0.05*).

	**16S rRNA**		**18S rRNA**	
**PERMANOVA**	**p-value**	**t-statistic**	**p-value**	**t-statistic**
Genuine/Falsified	***0.001***	*4.213*	***0.001***	*3.662*
Packaging Type	***0.001***	*3.389*	***0.001***	*3.662*
Sample	***0.001***	*2.931*	***0.001***	*2.286*
**Faith_PD**	**p-value**	**H-value**	**p-value**	**H-value**
Genuine/Falsified	*0.047*	*3.927*	***0.0003***	*12.870*
Packaging Type	*0.250*	*4.112*	***0.00003***	*23.787*
Sample	***0.003***	*26.363*	***0.0002***	*34.171*
**Number of Reads**	**p-value**	**F-statistic**	**p-value**	**F-statistic**
Genuine/Falsified	*0.1258*	*2.398*	***0.009***	*7.121*
Packaging Type	*0.3058*	*1.217*	*0.870*	*0.1397*
Sample	***0.00019***	*4.15*	***0.01781***	*2.384*

Table S2: Pairwise p-values from PERMANOVA of Bray-Curtis Distance Matrix for 16S rRNA data. n=number of tablets per sample included in analysis. Tablet samples with no statistically significant differences (*p<0.05*) are in Bold.

	2/15009	2/15011	2/15044	2/15058	China07/10	G015	G218	G220	Lao05/17	Lao07/04
2/15057 (n=5)	*0.008*	*0.001*	*0.03*	***0.068***	*0.018*	*0.001*	*0.001*	*0.002*	*0.001*	*0.001*
2/15009 (n=4)		***0.628***	*0.003*	*0.003*	*0.028*	*0.001*	*0.001*	*0.005*	*0.002*	*0.002*
2/15011 (n=4)			*0.001*	*0.001*	*0.029*	*0.001*	*0.002*	*0.003*	*0.002*	*0.002*
2/15044 (n=4)	.			*0.001*	*0.028*	*0.002*	*0.002*	*0.002*	*0.005*	*0.004*
2/15058 (n=5)					*0.019*	*0.001*	*0.002*	*0.001*	*0.002*	*0.002*
China (n=1)						*0.014*	***0.078***	***0.063***	***0.059***	***0.05***
G015 (n=5)							*0.002*	*0.002*	*0.001*	*0.001*
G218 (n=2)								***0.088***	*0.025*	*0.004*
G220 (n=2)									*0.029*	*0.007*
Lao_05/17 (n=2)										*0.005*

Table S3: Pairwise p-values from PERMANOVA of Bray-Curtis Distance Matrix for 18S rRNA data. n=number of tablets per sample included in analysis. Tablet samples with no statistically significant differences ($p<0.05$) are highlighted in Bold.

	2/15009	*2/15011*	*2/15044*	*2/15058*	*China07/10*	*G015*	*G218*	*G220*	*Lao05/17*	*Lao07/04*
2/15057 (n=5)	*0.001*	*0.001*	***0.32***	*0.003*	***0.058***	*0.005*	*0.006*	*0.002*	*0.003*	*0.001*
2/15009 (n=4)		***0.956***	*0.001*	***0.412***	*0.017*	*0.001*	*0.001*	*0.002*	*0.004*	*0.001*
2/15011 (n=4)			*0.001*	*0.003*	*0.028*	*0.001*	*0.001*	*0.002*	*0.004*	*0.001*
2/15044 (n=4)				*0.001*	*0.039*	***0.076***	*0.004*	*0.002*	*0.001*	*0.001*
2/15058 (n=5)					*0.017*	*0.001*	*0.003*	*0.003*	*0.001*	*0.001*
China07/10 (n=1)						*0.034*	***0.062***	***0.077***	***0.056***	*0.023*
G015 (n=5)							*0.013*	*0.007*	*0.003*	*0.001*
G218 (n=2)								***0.253***	*0.029*	*0.002*
G220 (n=2)									*0.022*	*0.003*
Lao05/17 (n=2)										*0.006*

Table S4: Wilcoxon rank sum test/Mann-Whitney significance test of Bray-Curtis similarity 'Within' and 'Between' samples for 16S and 18S using genuine tablets only, falsified tablets only and all tablets combined.

		Number of samples		Median Bray-Curtis Similarity		Within Vs Between	Within Genuine vs within
		**Within**	**Between**	**Within**	**Between**	***p-value (W)***	***p-value (W)***
**16S**	**Genuine**	79	272	0.227	0.055	*2.2e-16 (19074)*	NA
	**Falsified**	171	910	0.078	0.038	*4.519e-16 (107918)*	*2.2e-16 (11098)*
	**All**	250	2451	0.109	0.039	*2.2e-16 (472951)*	*1.886e-09 (14219)*
**18S**	**Genuine**	62	238	0.219	0.063	*1.229e-10 (11227)*	NA
	**Falsified**	163	872	0.088	0.038	*9.813e-11 (93299)*	*9.294e08 (7326.5)*
	**All**	225	2260	0.112	0.038	*2.2e-16 (356588)*	*4.242e-5 (9248.5)*

Table S5: Eukaryote species identified as indicator taxa (differentially abundant) for genuine and falsified tablets. Positive log2FoldChange (G/F) values indicate 'genuine' indicator taxa and negative log2FoldChange (G/F) values indicate 'falsified' indicator taxa.

Indicator species	log2FoldChange	mean_rel_abu Falsified	mean_rel_abun Genuine
**Genuine**			
Piskurozyma capsuligena	2.108	0.009	0.056
Scheffersomyces coipomoensis	2.439	0.011	0.106
**Falsified**			
Epistylis hentscheli	-3.457	0.076	0.017
Ambomucor seriatoinflatus	-2.965	0.184	0.049
Placus salinus	-2.922	0.100	0.018
Gerris buenoi	-2.867	0.043	0.013
Dictyochloropsis asterochloroides	-2.848	0.051	0.013
Salpingoeca fusiformis	-2.808	0.139	0.034
Allovahlkampfia sp. PKD 2011a	-2.766	0.142	0.031
Cyanidioschyzon sp. Y16	-2.678	0.074	0.013
Ichthyobodo necator	-2.577	0.073	0.013
Apicomplexa sp. 1 KCW-2013	-2.516	0.069	0.013

Table S6: Eukaryote ASVs identified as indicator taxa (differentially abundant) for packaging Type 8 or 11. Positive log2FoldChange values indicate 'Falsified Type 8' indicator taxa and negative log2FoldChange values indicate 'Falsified Type 11' indicator taxa. Letters in italics indicate the lowest taxonomic level an ASV could be identified: Phylum (*p*), Class (*c*), subclass (*sc*), Order (*o*), Family (*f*), and Genus (*g*). ASVs identified to species level are highlighted in bold.

Taxa	Packaging Type	Indicator Taxa	log2FoldChange
**Fungi**	**8**	Saccharomycetales *o*	3.311029692
		Eurotiomycetes *c*	2.716040366
		uncultured Chytridiomycota *p*	2.463027639
		Saccharomycetales *o*	2.458922543
		Agaricomycetes *c*	2.329453251
	**11**	Agaricomycetes *c*	-3.883331435
		Saccharomycetales *o*	-3.583393581
		Herpotrichiellaceae *f*	-3.426031565
		***Pichia* sp.**	-2.992190399
		Saccharomycetales *o*	-2.57834746
**Algae**	**8**	Chrysophyceae *c*	2.38583785
		Chlorophyceae *c*	2.87853893
	**11**	Chlorophyceae *c*	-2.857943112
		Dictyochloropsis asterochloroides	-2.735711662
		Cladophorales *o*	-2.717944156
**Ciliophora**	**8**	Haptoria *sc*	4.639548101
		Hymenostomatia *o*	3.049315105
		Hymenostomatia *o*	2.572686307
		Strobilidium caudatum	2.514828777
		Epistylis *g*	2.452762119
		Colpodida *o*	2.370996752
	**11**	uncultured Halteria *g*	-3.454260373
		Hymenostomatia *o*	-3.382368
		Hymenostomatia *o*	-3.347008616
		Tetrahymena *g*	-3.056295144
		Synura sp. HCB-2005	-2.738455866
		Spirostomum *g*	-2.514256851

		Hypotrichia [g]	-2.432196867
**Diatoms**	**8**	Bacillariophyceae [p]	2.648950934
	**11**	Bacillariophyceae [p]<br>Bacillariophyceae [p]	-3.458797784<br>-3.258257002
**Other**	**8**	Naegleria *(genus of amoebae protist)*<br>Magnoliophyta *(Clade of seed plant)*	2.769694432<br>2.291942869
	**11**	*Neobodo (genus of protists)*<br>uncultured katablepharid *(Order of heterotrophic flagellates)*	-4.112716466<br>-2.295241965

Table S7: Details the lysis protocol, MiSeq run, forward and reverse barcode combinations and P5/P7 index used for all DNA extracts analysed by 16S and 18S rRNA.

					16S Bacteria							18S Eukaryotes						
Tablet Batch ID	Tablet ID	DNA Extract ID	DNA Extraction Protocol/ Batch	MiSeq Run	Forward Barcode	Reverse Barcode	Adapter Ligation Pool	P7 Index	P7 index Sequence	P5 Index	P5 index Sequence	Forward Barcode	Reverse Barcode	Adapter Ligation Pool	P7 Index	P7 index Sequence	P5 Index	P5 index Sequence
2/15044	9_1	N9-1A	A1	R1	ACAGCTG	TGTACGTG	2	P1	AACAACCG	NA	NA	AGAGAC	TAGCACG	2	P3	TTCCTCCT	NA	NA
2/15044	9_1	N9-1B	B1	R1	ACAGCTG	TGACGTGG	2	P1	AACAACCG	NA	NA	TAGCGTCG	TCTACTCG	2	P3	TTCCTCCT	NA	NA
2/15044	9_2	N9-2A	A1	R1	ACAGCTG	ACAGCTGG	1	P1	AACAACCG	NA	NA	TGAGACG	AAGCAG	1	P3	TTCCTCCT	NA	NA
2/15044	9_2	N9-2B	B1	R1	ACAGCTG	ATCGTCTG	1	P1	AACAACCG	NA	NA	TAGCACG	AGAGAC	1	P3	TTCCTCCT	NA	NA
2/15044	9_3	N9-3A	A1	R1	ACAGCTG	TAGCTCTG	2	P1	AACAACCG	NA	NA	TCTACTCG	TAGCGTCG	1	P3	TTCCTCCT	NA	NA
2/15044	9_3	N9-3B	B1	R1	ATCGTCT	TCTCTGTG	1	P1	AACAACCG	NA	NA	AAGCAG	TAGCACG	2	P3	TTCCTCCT	NA	NA
15057	10_1	N10-1A	A1	R1	TGTACGT	TGTACGTG	2	P1	AACAACCG	NA	NA	AGAGAC	TAGCGTCG	2	P3	TTCCTCCT	NA	NA
15057	10_1	N10-1B	B1	R1	TGTACGT	TGACGTGG	2	P1	AACAACCG	NA	NA	TAGCGTCG	TGAGACG	2	P3	TTCCTCCT	NA	NA
15057	10_2	N10-2A	A1	R1	TGTACGT	ACAGCTGG	1	P1	AACAACCG	NA	NA	TGAGACG	TAGCACG	2	P3	TTCCTCCT	NA	NA
15057	10_2	N10-2B	B1	R1	TGTACGT	ATCGTCTG	1	P1	AACAACCG	NA	NA	TAGCACG	TCTACTCG	1	P3	TTCCTCCT	NA	NA
15057	10_3	N10-3A	A1	R1	TGTACGT	TAGCTCTG	1	P1	AACAACCG	NA	NA	TCTACTCG	AAGCAG	1	P3	TTCCTCCT	NA	NA
15057	10_3	N10-3B	B1	R1	TGACGTG	TCTCTGTG	1	P1	AACAACCG	NA	NA	AAGCAG	TAGCGTCG	2	P3	TTCCTCCT	NA	NA
G015	14_1	N14-1A	A1	R1	TGACGTG	TGTACGTG	2	P1	AACAACCG	NA	NA	AGAGAC	TGAGACG	2	P3	TTCCTCCT	NA	NA
G015	14_1	N14-1B	B1	R1	TGACGTG	TGACGTGG	2	P1	AACAACCG	NA	NA	TAGCGTCG	TAGCACG	2	P3	TTCCTCCT	NA	NA
G015	14_2	N14-2A	A1	R1	TGACGTG	ACAGCTGG	2	P1	AACAACCG	NA	NA	TGAGACG	TCTACTCG	1	P3	TTCCTCCT	NA	NA
G015	14_2	N14-2B	B1	R1	TGACGTG	ATCGTCTG	2	P1	AACAACCG	NA	NA	TAGCACG	AAGCAG	1	P3	TTCCTCCT	NA	NA
G015	14_3	N14-3A	A1	R1	TGACGTG	TAGCTCTG	2	P1	AACAACCG	NA	NA	TCTACTCG	AGAGAC	1	P3	TTCCTCCT	NA	NA
G015	14_3	N14-3B	B1	R1	ACAGCTG	TCTCTGTG	2	P1	AACAACCG	NA	NA	AAGCAG	TGAGACG	1	P3	TTCCTCCT	NA	NA
EBC	NA	EBC-A1	A1	R1	ATCGTCT	TGTACGTG	2	P1	AACAACCG	NA	NA	AGAGAC	TCTACTCG	2	P3	TTCCTCCT	NA	NA
EBC	NA	EBC-A2	A1	R1	ATCGTCT	ACAGCTGG	1	P1	AACAACCG	NA	NA	TAGCGTCG	AAGCAG	2	P3	TTCCTCCT	NA	NA
EBC	NA	EBC-B1	B1	R1	ATCGTCT	TGACGTGG	1	P1	AACAACCG	NA	NA	TGAGACG	AGAGAC	2	P3	TTCCTCCT	NA	NA
EBC	NA	EBC-B2	B1	R1	ATCGTCT	ATCGTCTG	1	P1	AACAACCG	NA	NA	TAGCACG	TAGCGTCG	1	P3	TTCCTCCT	NA	NA
NTC	NA	NTC	NA	R1	TCTCTGT	TCTCTGTG	1	P1	AACAACCG	NA	NA	AAGCAG	AAGCAG	1	P3	TTCCTCCT	NA	NA
Lao_05/17	2_1	2-1A	A2	R2	ACTCACTG	TCTCTGTG	1	P1	AACAACCG	P1	AACAACCG	AAGCAG	AAGCAG	1	P1	AACAACCG	P4	GCTACAAC
Lao_05/17	2_1	2-1B	B2	R2	ACTCACTG	TGTACGTG	1	P1	AACAACCG	P1	AACAACCG	AGAGAC	AAGCAG	1	P1	AACAACCG	P4	GCTACAAC
Lao_05/17	2_2	2-2A	A3	R2	ACTCACTG	TGACGTGG	1	P1	AACAACCG	P1	AACAACCG	TAGCGTCG	AAGCAG	1	P1	AACAACCG	P4	GCTACAAC
Lao_05/17	2_2	2-2B	B3	R2	ACTCACTG	ACAGCTGG	1	P1	AACAACCG	P1	AACAACCG	TGAGACG	AAGCAG	1	P1	AACAACCG	P4	GCTACAAC
Lao_07/04	5_1	5-1A	A2	R2	ACTCACTG	ATCGTCTG	1	P1	AACAACCG	P1	AACAACCG	TAGCACG	AAGCAG	1	P1	AACAACCG	P4	GCTACAAC
Lao_07/04	5_1	5-1B	B2	R2	ACTCACTG	TAGCTCTG	1	P1	AACAACCG	P1	AACAACCG	TCTACTCG	AAGCAG	1	P1	AACAACCG	P4	GCTACAAC
Lao_07/04	5_2	5-2A	A3	R2	ACTCACTG	AGCACTGG	1	P1	AACAACCG	P1	AACAACCG	ATGACTCG	AAGCAG	1	P1	AACAACCG	P4	GCTACAAC
Lao_07/04	5_2	5-2B	B3	R2	ACTCACTG	TACTATGG	1	P1	AACAACCG	P1	AACAACCG	ACACACG	AAGCAG	1	P1	AACAACCG	P4	GCTACAAC
Lao_07/04	5_3	5-3A	A2	R2	ACTCACTG	AGTATCTG	1	P1	AACAACCG	P1	AACAACCG	AGAGTAG	AAGCAG	1	P1	AACAACCG	P4	GCTACAAC
Lao_07/04	5_3	5-3B	B2	R2	ACTCACTG	TCGAGCTG	1	P1	AACAACCG	P1	AACAACCG	ATCTATCG	AAGCAG	1	P1	AACAACCG	P4	GCTACAAC
Lao_07/04	5_4	5-4A	A3	R2	ACTCACTG	AACCGAGT	1	P1	AACAACCG	P1	AACAACCG	ACAGATCG	AAGCAG	1	P1	AACAACCG	P4	GCTACAAC
Lao_07/04	5_4	5-4B	B3	R2	ACTCACTG	TAGAGCGT	1	P1	AACAACCG	P1	AACAACCG	AAGCAG	AGAGAC	1	P1	AACAACCG	P4	GCTACAAC
China_07/10	6_1	6-1A	A3	R2	ACTCACTG	TCTGATGG	1	P1	AACAACCG	P1	AACAACCG	AGAGAC	AGAGAC	1	P1	AACAACCG	P4	GCTACAAC
China_07/10	6_1	6-1B	B3	R2	ACTCACTG	ATGCATGG	1	P1	AACAACCG	P1	AACAACCG	TAGCGTCG	AGAGAC	1	P1	AACAACCG	P4	GCTACAAC
2/15044	9_4	9-4A	A2	R2	ACTCACTG	TCATACTG	1	P1	AACAACCG	P1	AACAACCG	TGAGACG	AGAGAC	1	P1	AACAACCG	P4	GCTACAAC
2/15044	9_4	9-4B	B2	R2	ACTCACTG	TACGACTG	1	P1	AACAACCG	P1	AACAACCG	TAGCACG	AGAGAC	1	P1	AACAACCG	P4	GCTACAAC
15057	10_4	10-4A	A2	R2	AGAGTATG	TCTCTGTG	1	P1	AACAACCG	P1	AACAACCG	TCTACTCG	AGAGAC	1	P1	AACAACCG	P4	GCTACAAC
15057	10_4	10-4B	B2	R2	AGAGTATG	TGTACGTG	1	P1	AACAACCG	P1	AACAACCG	ATGACTCG	AGAGAC	1	P1	AACAACCG	P4	GCTACAAC
15057	10_5	10-5A	A3	R2	AGAGTATG	TGACGTGG	1	P1	AACAACCG	P1	AACAACCG	ACACACG	AGAGAC	1	P1	AACAACCG	P4	GCTACAAC
15057	10_5	10-5B	B3	R2	AGAGTATG	ACAGCTGG	1	P1	AACAACCG	P1	AACAACCG	AGAGTAG	AGAGAC	1	P1	AACAACCG	P4	GCTACAAC
2/15058	11_1	11-1A	A2	R2	AGAGTATG	ATCGTCTG	2	P2	CACGTCTA	P2	CACGTCTA	ATCTATCG	AGAGAC	2	P2	CACGTCTA	P5	CGACACTT
2/15058	11_1	11-1B	B2	R2	AGAGTATG	TAGCTCTG	2	P2	CACGTCTA	P2	CACGTCTA	ACAGATCG	AGAGAC	1	P1	AACAACCG	P4	GCTACAAC
2/15058	11_2	11-2A	A2	R2	AGAGTATG	AGCACTGG	2	P2	CACGTCTA	P2	CACGTCTA	AAGCAG	TAGCGTCG	1	P1	AACAACCG	P4	GCTACAAC
2/15058	11_2	11-2B	B2	R2	AGAGTATG	TACTATGG	2	P2	CACGTCTA	P2	CACGTCTA	AGAGAC	TAGCGTCG	1	P1	AACAACCG	P4	GCTACAAC
2/15058	11_3	11-3A	A2	R2	AGAGTATG	AGTATCTG	2	P2	CACGTCTA	P2	CACGTCTA	TAGCGTCG	TAGCGTCG	2	P2	CACGTCTA	P5	CGACACTT
2/15058	11_3	11-3B	B2	R2	AGAGTATG	TCGAGCTG	2	P2	CACGTCTA	P2	CACGTCTA	TGAGACG	TAGCGTCG	2	P2	CACGTCTA	P5	CGACACTT
2/15058	11_4	11-4A	A3	R2	AGAGTATG	AACCGAGT	2	P2	CACGTCTA	P2	CACGTCTA	TAGCACG	TAGCGTCG	2	P2	CACGTCTA	P5	CGACACTT
2/15058	11_4	11-4B	B3	R2	AGAGTATG	TAGAGCGT	2	P2	CACGTCTA	P2	CACGTCTA	TCTACTCG	TAGCGTCG	2	P2	CACGTCTA	P5	CGACACTT
2/15058	11_5	11-5A	A3	R2	AGAGTATG	TCTGATGG	2	P2	CACGTCTA	P2	CACGTCTA	ATGACTCG	TAGCGTCG	2	P2	CACGTCTA	P5	CGACACTT
2/15058	11_5	11-5B	B3	R2	AGAGTATG	ATGCATGG	2	P2	CACGTCTA	P2	CACGTCTA	ACACACG	TAGCGTCG	2	P2	CACGTCTA	P5	CGACACTT
2/15009	12_1	12-1A	A2	R2	AGAGTATG	TCATACTG	2	P2	CACGTCTA	P2	CACGTCTA	AGAGTAG	TAGCGTCG	2	P2	CACGTCTA	P5	CGACACTT
2/15009	12_1	12-1B	B2	R2	AGAGTATG	TACGACTG	2	P2	CACGTCTA	P2	CACGTCTA	ATCTATCG	TAGCGTCG	2	P2	CACGTCTA	P5	CGACACTT
2/15009	12_2	12-2A	A3	R2	TGTCTCGG	TCTCTGTG	2	P2	CACGTCTA	P2	CACGTCTA	ACAGATCG	TAGCGTCG	2	P2	CACGTCTA	P5	CGACACTT
2/15009	12_2	12-2B	B3	R2	TGTCTCGG	TGTACGTG	2	P2	CACGTCTA	P2	CACGTCTA	AAGCAG	TGAGACG	2	P2	CACGTCTA	P5	CGACACTT
2/15009	12_3	12-3A	A2	R2	TGTCTCGG	TGACGTGG	2	P2	CACGTCTA	P2	CACGTCTA	AGAGAC	TGAGACG	2	P2	CACGTCTA	P5	CGACACTT
2/15009	12_3	12-3B	B2	R2	TGTCTCGG	ACAGCTGG	2	P2	CACGTCTA	P2	CACGTCTA	TAGCGTCG	TGAGACG	2	P2	CACGTCTA	P5	CGACACTT
2/15009	12_4	12-4A	A3	R2	TGTCTCGG	ATCGTCTG	2	P2	CACGTCTA	P2	CACGTCTA	TGAGACG	TGAGACG	2	P2	CACGTCTA	P5	CGACACTT
2/15009	12_4	12-4B	B3	R2	TGTCTCGG	TAGCTCTG	2	P2	CACGTCTA	P2	CACGTCTA	TAGCACG	TGAGACG	2	P2	CACGTCTA	P5	CGACACTT
2/15011	13_1	13-1A	A2	R2	TGTCTCGG	AGCACTGG	2	P2	CACGTCTA	P2	CACGTCTA	TCTACTCG	TGAGACG	2	P2	CACGTCTA	P5	CGACACTT
2/15011	13_1	13-1B	B2	R2	TGTCTCGG	TACTATGG	2	P2	CACGTCTA	P2	CACGTCTA	ATGACTCG	TGAGACG	2	P2	CACGTCTA	P5	CGACACTT
2/15011	13_2	13-2A	A3	R2	TGTCTCGG	AGTATCTG	3	P3	TTCCTCCT	P3	TTCCTCCT	ACACACG	TGAGACG	3	P3	TTCCTCCT	P6	GATCTTGC
2/15011	13_2	13-2B	B3	R2	TGTCTCGG	TCGAGCTG	3	P3	TTCCTCCT	P3	TTCCTCCT	AGAGTAG	TGAGACG	3	P3	TTCCTCCT	P6	GATCTTGC
2/15011	13_3	13-3A	A2	R2	TGTCTCGG	AACCGAGT	3	P3	TTCCTCCT	P3	TTCCTCCT	ATCTATCG	TGAGACG	3	P3	TTCCTCCT	P6	GATCTTGC
2/15011	13_3	13-3B	B2	R2	TGTCTCGG	TAGAGCGT	3	P3	TTCCTCCT	P3	TTCCTCCT	ACAGATCG	TGAGACG	3	P3	TTCCTCCT	P6	GATCTTGC
2/15011	13_4	13-4A	A3	R2	TGTCTCGG	TCTGATGG	3	P3	TTCCTCCT	P3	TTCCTCCT	AAGCAG	TAGCACG	3	P3	TTCCTCCT	P6	GATCTTGC
2/15011	13_4	13-4B	B3	R2	TGTCTCGG	ATGCATGG	3	P3	TTCCTCCT	P3	TTCCTCCT	AGAGAC	TAGCACG	3	P3	TTCCTCCT	P6	GATCTTGC
G015	14_4	14-4A	A3	R2	TGTCTCGG	TCATACTG	3	P3	TTCCTCCT	P3	TTCCTCCT	TAGCGTCG	TAGCACG	3	P3	TTCCTCCT	P6	GATCTTGC
G015	14_4	14-4B	B3	R2	TGTCTCGG	TACGACTG	3	P3	TTCCTCCT	P3	TTCCTCCT	TGAGACG	TAGCACG	3	P3	TTCCTCCT	P6	GATCTTGC
G015	14_5	14-5A	A2	R2	AGCTGCGG	TCTCTGTG	3	P3	TTCCTCCT	P3	TTCCTCCT	TAGCACG	TAGCACG	3	P3	TTCCTCCT	P6	GATCTTGC
G015	14_5	14-5B	B2	R2	AGCTGCGG	TGTACGTG	3	P3	TTCCTCCT	P3	TTCCTCCT	TCTACTCG	TAGCACG	3	P3	TTCCTCCT	P6	GATCTTGC
G218	15_1	15-1A	A2	R2	AGCTGCGG	TGACGTGG	3	P3	TTCCTCCT	P3	TTCCTCCT	ATGACTCG	TAGCACG	3	P3	TTCCTCCT	P6	GATCTTGC
G218	15_1	15-1B	B2	R2	AGCTGCGG	ACAGCTGG	3	P3	TTCCTCCT	P3	TTCCTCCT	ACACACG	TAGCACG	3	P3	TTCCTCCT	P6	GATCTTGC
G218	15_2	15-2A	A3	R2	AGCTGCGG	ATCGTCTG	3	P3	TTCCTCCT	P3	TTCCTCCT	AGAGTAG	TAGCACG	3	P3	TTCCTCCT	P6	GATCTTGC
G218	15_2	15-2B	B3	R2	AGCTGCGG	TAGCTCTG	3	P3	TTCCTCCT	P3	TTCCTCCT	ATCTATCG	TAGCACG	3	P3	TTCCTCCT	P6	GATCTTGC
G220	16_1	16-1A	A2	R2	AGCTGCGG	AGCACTGG	3	P3	TTCCTCCT	P3	TTCCTCCT	ACAGATCG	TCTACTCG	3	P3	TTCCTCCT	P6	GATCTTGC
G220	16_1	16-1B	B2	R2	AGCTGCGG	TACTATGG	3	P3	TTCCTCCT	P3	TTCCTCCT	AAGCAG	TCTACTCG	3	P3	TTCCTCCT	P6	GATCTTGC
G220	16_2	16-2A	A3	R2	AGCTGCGG	AGTATCTG	3	P3	TTCCTCCT	P3	TTCCTCCT	AGAGAC	TCTACTCG	3	P3	TTCCTCCT	P6	GATCTTGC
G220	16_2	16-2B	B3	R2	AGCTGCGG	TCGAGCTG	3	P3	TTCCTCCT	P3	TTCCTCCT	TAGCGTCG	TCTACTCG	3	P3	TTCCTCCT	P6	GATCTTGC
EBC	NA	EBC-1A	A2	R2	AGCTGCGG	AACCGAGT	3	P3	TTCCTCCT	P3	TTCCTCCT	TGAGACG	TCTACTCG	3	P3	TTCCTCCT	P6	GATCTTGC
EBC	NA	EBC-1B	B2	R2	AGCTGCGG	TAGAGCGT	3	P3	TTCCTCCT	P3	TTCCTCCT	TAGCACG	TCTACTCG	3	P3	TTCCTCCT	P6	GATCTTGC
EBC	NA	EBC-2A	A3	R2	AGCTGCGG	TCTGATGG	3	P3	TTCCTCCT	P3	TTCCTCCT	TCTACTCG	TCTACTCG	3	P3	TTCCTCCT	P6	GATCTTGC
EBC	NA	EBC-2B	B3	R2	AGCTGCGG	ATGCATGG	3	P3	TTCCTCCT	P3	TTCCTCCT	ATGACTCG	TCTACTCG	3	P3	TTCCTCCT	P6	GATCTTGC

Table S8: Summary of the number of 16S rRNA sequence reads obtained per DNA extract following each data processing step.

Tablet Batch ID	DNA Extract ID	# Sequences Demultiplex and Trim	# Sequences after Quality Filter (deblur)	% Sequences Retained	# Sequences after Control filtering	% Reads Retained
Lao_05/17	2-1A	10130	6,205	61.3	4,253	42.0
Lao_05/17	2-1B	10954	6,793	62.0	3,271	29.9
Lao_05/17	2-2A	14047	8,587	61.1	6,021	42.9
Lao_05/17	2-2B	10581	6,668	63.0	4,137	39.1
Lao_07/04	5-1A	9746	6,328	64.9	2,226	22.8
Lao_07/04	5-1B	19161	11,887	62.0	3,907	20.4
Lao_07/04	5-2A	8884	5,782	65.1	2,170	24.4
Lao_07/04	5-2B	8082	5,094	63.0	2,770	34.3
Lao_07/04	5-3A	7663	4,528	59.1	1,635	21.3
Lao_07/04	5-3B	3583	2,044	57.0	866	24.2
Lao_07/04	5-4A	8822	5,743	65.1	2,745	31.1
Lao_07/04	5-4B	7437	4,518	60.8	1,829	24.6
China_07/10	6-1A	6715	4,287	63.8	2,100	31.3
China_07/10	6-1B	8898	5,979	67.2	3,676	41.3
2/15044	9-1A	74832	62,008	82.9	58,699	78.4
2/15044	9-1B	230347	176,391	76.6	159,258	69.1
2/15044	9-2A	54174	47,009	86.8	46,928	86.6
2/15044	9-2B	19078	15,700	82.3	15,179	79.6
2/15044	9-3A	75208	56,182	74.7	54,547	72.5
2/15044	9-3B	57974	45,849	79.1	37,219	64.2
2/15044	9-4A	9601	3,874	40.3	2,538	26.4
2/15044	9-4B	7090	4,217	59.5	2,079	29.3
15057	10-1A	35212	27,826	79.0	23,622	67.1
15057	10-1B	119291	92,933	77.9	85,411	71.6
15057	10-2A	60897	47,488	78.0	46,163	75.8
15057	10-2B	115220	93,195	80.9	82,598	71.7
15057	10-3A	3677	2,767	75.3	2,754	74.9
G015	10-3B	19092	16,173	84.7	15,128	79.2
15057	10-4A	6088	3,587	58.9	2,327	38.2
15057	10-4B	7253	4,499	62.0	2,262	31.2
15057	10-5A	10671	6,136	57.5	4,546	42.6
15057	10-5B	9776	6,198	63.4	4,429	45.3
2/15058	11-1A	21181	13,645	64.4	8,182	38.6
2/15058	11-1B	17464	11,097	63.5	4,114	23.6
2/15058	11-2A	27116	17,020	62.8	10,596	39.1
2/15058	11-2B	18690	12,157	65.0	7,222	38.6
2/15058	11-3A	16863	10,516	62.4	7,751	46.0
2/15058	11-3B	7876	4,924	62.5	2,737	34.8
2/15058	11-4A	17860	10,617	59.4	8,782	49.2
2/15058	11-4B	19937	13,993	70.2	7,101	35.6
2/15058	11-5A	14523	9,024	62.1	7,518	51.8
2/15058	11-5B	17195	11,583	67.4	3,756	21.8
2/15009	12-1A	17380	10,861	62.5	6,208	35.7
2/15009	12-1B	18177	11,593	63.8	6,329	34.8
2/15009	12-2A	14980	11,949	79.8	6,150	41.1
2/15009	12-2B	16147	12,330	76.4	4,308	26.7
2/15009	12-3A	27351	20,631	75.4	10,698	39.1
2/15009	12-3B	22523	16,318	72.5	8,560	38.0
2/15009	12-4A	23800	18,435	77.5	9,023	37.9
2/15009	12-4B	20279	14,874	73.3	2,606	12.9
2/15011	13-1A	28909	20,516	71.0	10,378	35.9
2/15011	13-1B	22613	16,246	71.8	8,543	37.8
2/15011	13-2A	56025	42,734	76.3	21,010	37.5
2/15011	13-2B	9334	7,013	75.1	2,802	30.0
2/15011	13-3A	24938	18,867	75.7	11,775	47.2
2/15011	13-3B	23003	16,749	72.8	7,858	34.2
2/15011	13-4A	16215	11,941	73.6	6,204	38.3
2/15011	13-4B	20281	15,401	75.9	6,004	29.6
G015	14-1A	143616	115,558	80.5	103,780	72.3
G015	14-1B	7541	5,279	70.0	5,001	66.3
G015	14-2A	229346	171,866	74.9	162,083	70.7
G015	14-2B	277762	221,801	79.9	214,488	77.2
G015	14-3A	83013	61,010	73.5	59,484	71.7
2/15044?	14-3B	52407	42,333	80.8	39,580	75.5
G015	14-4A	17661	12,857	72.8	4,518	25.6
G015	14-4B	18127	13,033	71.9	4,572	25.2
G015	14-5A	18541	14,080	75.9	5,096	27.5
G015	14-5B	20214	14,386	71.2	4,640	23.0
G218	15-1A	29658	21,329	71.9	3,930	13.3
G218	15-1B	27159	18,859	69.4	4,130	15.2
G218	15-2A	21871	15,854	72.5	2,792	12.8
G218	15-2B	24176	17,586	72.7	4,278	17.7
G220	16-1A	26497	18,797	70.9	3,703	14.0
G220	16-1B	18921	14,996	79.3	4,011	21.2
G220	16-2A	30479	21,579	70.8	4,958	16.3
G220	16-2B	10995	7,854	71.4	1,451	13.2
NA	EBC-1A	25564	21,468	84.0	NA	NA
NA	EBC-1B	23067	18,550	80.4	NA	NA
NA	EBC-2A	12603	9,752	77.4	NA	NA
NA	EBC-2B	17673	14,564	82.4	NA	NA
NA	EBC-A1	155848	118,774	76.2	NA	NA
NA	EBC-A2	20702	16,528	79.8	NA	NA
NA	EBC-B1	324775	247,414	76.2	NA	NA
NA	EBC-B2	24559	19,769	80.5	NA	NA
	PCRNEG1	124	67	54.0	NA	NA

Table S9: Summary of the number of 18S rRNA sequence reads obtained per DNA extract following each data processing step.

Tablet Batch ID	DNA Extract ID	# Sequences Demultiplex and Trim	# Sequences after Quality Filter (dada2)	% Sequences Retained	# Sequences after Control filtering	% Reads Retained
Lao_05/17	2-1A	26955	25,198	93.5	16,037	59.50
Lao_05/17	2-1B	24488	24,068	98.3	10,457	42.70
Lao_05/17	2-2A	8157	7,939	97.3	5,605	68.71
Lao_05/17	2-2B	16431	16,381	99.7	11,309	68.83
Lao_07/04	5-1A	11145	11,053	99.2	2,606	23.38
Lao_07/04	5-1B	8202	8,160	99.5	2,051	25.01
Lao_07/04	5-2A	33152	32,354	97.6	10,683	32.22
Lao_07/04	5-2B	15791	15,761	99.8	7,032	44.53
Lao_07/04	5-3A	15053	15,000	99.6	4,700	31.22
Lao_07/04	5-3B	15742	15,611	99.2	4,088	25.97
Lao_07/04	5-4A	25077	24,637	98.2	8,093	32.27
Lao_07/04	5-4B	43922	42,763	97.4	15,026	34.21
China_07/10	6-1A	43471	42,512	97.8	20,632	47.46
China_07/10	6-1B	20375	20,264	99.5	9,997	49.07
2/15044	9-1A	841	722	85.9	230	27.35
2/15044	9-1B	251469	14,403	5.7	8,490	3.38
2/15044	9-2A	88052	985	1.1	520	0.59
2/15044	9-2B	63844	171	0.3	162	0.25
2/15044	9-3A	233719	206,037	88.2	74,922	32.06
2/15044	9-3B	303	259	85.5	160	52.81
2/15044	9-4A	30274	29,721	98.2	11,021	36.40
2/15044	9-4B	22993	22,687	98.7	6,147	26.73
15057	10-1A	51551	50,581	98.1	13,027	25.27
15057	10-1B	20799	20,236	97.3	5,129	24.66
15057	10-2A	2658	272	10.2	12	0.45
15057	10-2B	23251	21,438	92.2	8,633	37.13
15057	10-3A	4086	175	4.3	26	0.64
15057	10-3B	56768	55,284	97.4	29,904	52.68
15057	10-4A	19087	18,959	99.3	5,526	28.95
15057	10-4B	119910	116,152	96.9	32,411	27.03
15057	10-5A	28980	28,803	99.4	14,717	50.78
15057	10-5B	31388	31,203	99.4	15,857	50.52
2/15058	11-1A	110575	107,474	97.2	27,791	25.13
2/15058	11-1B	90803	88,636	97.6	28,551	31.44
2/15058	11-2A	19893	19,785	99.5	8,324	41.84
2/15058	11-2B	22027	21,820	99.1	7,104	32.25
2/15058	11-3A	13299	6,715	50.5	2,310	17.37
2/15058	11-3B	23336	23,141	99.2	9,375	40.17
2/15058	11-4A	17424	17,309	99.3	10,310	59.17
2/15058	11-4B	33884	17,987	53.1	9,297	27.44
2/15058	11-5A	32724	32,423	99.1	14,710	44.95
2/15058	11-5B	23388	23,241	99.4	14,655	62.66
2/15009	12-1A	21697	21,587	99.5	7,413	34.17
2/15009	12-1B	50591	49,351	97.5	18,006	35.59
2/15009	12-2A	18650	18,604	99.8	9,631	51.64
2/15009	12-2B	31500	31,395	99.7	17,868	56.72
2/15009	12-3A	33601	32,768	97.5	9,935	29.57
2/15009	12-3B	22823	22,299	97.7	5,629	24.66
2/15009	12-4A	38567	38,252	99.2	17,239	44.70
2/15009	12-4B	30802	30,378	98.6	15,601	50.65
2/15011	13-1A	24229	23,878	98.6	7,482	30.88
2/15011	13-1B	41548	40,375	97.2	12,024	28.94
2/15011	13-2A	26774	26,454	98.8	13,581	50.72
2/15011	13-2B	35882	35,645	99.3	14,799	41.24
2/15011	13-3A	75266	72,855	96.8	22,406	29.77
2/15011	13-3B	29993	29,233	97.5	8,493	28.32
2/15011	13-4A	28598	27,958	97.8	9,475	33.13
2/15011	13-4B	31097	30,409	97.8	13,573	43.65
G015	14-1A	2399	1,705	71.1	312	13.01
G015	14-1B	23418	121	0.5	0	0.00
G015	14-2A	36858	25,176	68.3	12,639	34.29
G015	14-2B	22296	85	0.4	85	0.38
G015	14-3A	30842	90	0.3	3	0.01
G015	14-3B	1624	1,402	86.3	396	24.38
G015	14-4A	20780	20,276	97.6	3,237	15.58
G015	14-4B	25495	25,125	98.5	5,704	22.37
G015	14-5A	18208	17,781	97.7	3,583	19.68
G015	14-5B	15833	15,587	98.4	2,799	17.68
G218	15-1A	26829	25,524	95.1	2,083	7.76
G218	15-1B	23283	22,549	96.8	2,730	11.73
G218	15-2A	26198	24,719	94.4	3,070	11.72
G218	15-2B	25424	24,270	95.5	2,634	10.36
G220	16-1A	8664	8,424	97.2	631	7.28
G220	16-1B	21020	20,323	96.7	2,288	10.88
G220	16-2A	19817	18,736	94.5	2,490	12.56
G220	16-2B	31901	14,212	44.6	1,116	3.50
NA	EBC-1A	23002	22,811	99.2	NA	NA
NA	EBC-1B	20113	19,854	98.7	NA	NA
NA	EBC-2A	29135	14,113	48.4	NA	NA
NA	EBC-2B	25886	25,620	99.0	NA	NA
NA	EBC-A2	164777	661	0.4	NA	NA
NA	EBC-B1	48467	360	0.7	NA	NA
NA	EBC-B2	38241	24,768	64.8	NA	NA
NA	PCRNEG1	14	11	78.6	NA	NA

Table S10: Details of the barcodes and indexes used for the whole mitochondrial genome analysis.

Extract ID	Lib Prep Method	Extract Volume Used	Y-adapter Well#	Internal Barcode	MITO_HYBCAP POOL	P7 Index primer ID	P7 Index primer sequence	P5 Index Primer ID	P5 Index Primer ID sequence
10-4A	FS (Fragmentase)	26uL	A1	AACAACCG	1	P7_primer_Idx004	GCTACAAC	P5_primer_Idx010	ATCATGCG
10-4B	FS (Fragmentase)	26uL	B1	CACGTCTA	1	P7_primer_Idx004	GCTACAAC	P5_primer_Idx010	ATCATGCG
11-2A	FS (Fragmentase)	26uL	C1	TTCCTCCT	1	P7_primer_Idx004	GCTACAAC	P5_primer_Idx010	ATCATGCG
11-4B	FS (Fragmentase)	26uL	D1	GCTACAAC	1	P7_primer_Idx004	GCTACAAC	P5_primer_Idx010	ATCATGCG
12-1B	FS (Fragmentase)	26uL	E1	CGACACTT	1	P7_primer_Idx004	GCTACAAC	P5_primer_Idx010	ATCATGCG
12-2A	FS (Fragmentase)	26uL	F1	GATCTTGC	1	P7_primer_Idx004	GCTACAAC	P5_primer_Idx010	ATCATGCG
13-2A	FS (Fragmentase)	26uL	G1	TGCTTGCT	1	P7_primer_Idx004	GCTACAAC	P5_primer_Idx010	ATCATGCG
LIB_NEG_1	FS (Fragmentase)	26uL	H1	CCAACACT	1	P7_primer_Idx004	GCTACAAC	P5_primer_Idx010	ATCATGCG
10-4A	No Fragmentase/half volume	25ul	A2	CTAGCTCA	1	P7_primer_Idx004	GCTACAAC	P5_primer_Idx010	ATCATGCG
10-4B	No Fragmentase/half volume	25ul	B2	ATCATGCG	1	P7_primer_Idx004	GCTACAAC	P5_primer_Idx010	ATCATGCG
11-2A	No Fragmentase/half volume	25ul	C2	CAGCTAGA	1	P7_primer_Idx004	GCTACAAC	P5_primer_Idx010	ATCATGCG
11-4B	No Fragmentase/half volume	25ul	D2	TTGCGAGA	1	P7_primer_Idx004	GCTACAAC	P5_primer_Idx010	ATCATGCG
12-1B	No Fragmentase/half volume	25ul	E2	GGCGAATA	1	P7_primer_Idx004	GCTACAAC	P5_primer_Idx010	ATCATGCG
12-2A	No Fragmentase/half volume	25ul	F2	TACGACGT	1	P7_primer_Idx004	GCTACAAC	P5_primer_Idx010	ATCATGCG
13-2A	No Fragmentase/half volume	25ul	G2	CCAACTTC	1	P7_primer_Idx004	GCTACAAC	P5_primer_Idx010	ATCATGCG
LIB_NEG_2	No Fragmentase/half volume	25ul	H2	AGACATGC	1	P7_primer_Idx004	GCTACAAC	P5_primer_Idx010	ATCATGCG
11-1A	No Fragmentase/half volume	25ul	A3	CCTATTGG	1	P7_primer_Idx004	GCTACAAC	P5_primer_Idx010	ATCATGCG
11-1B	No Fragmentase/half volume	25ul	B3	TCTAGGAG	1	P7_primer_Idx004	GCTACAAC	P5_primer_Idx010	ATCATGCG
11-2B	No Fragmentase/half volume	25ul	C3	GATCTCAG	1	P7_primer_Idx004	GCTACAAC	P5_primer_Idx010	ATCATGCG
11-3A	No Fragmentase/half volume	25ul	D3	AACTTGCC	1	P7_primer_Idx004	GCTACAAC	P5_primer_Idx010	ATCATGCG
11-3B	No Fragmentase/half volume	25ul	E3	ACACGAGA	1	P7_primer_Idx004	GCTACAAC	P5_primer_Idx010	ATCATGCG
11-4A	No Fragmentase/half volume	25ul	F3	CATCCAAG	1	P7_primer_Idx004	GCTACAAC	P5_primer_Idx010	ATCATGCG
11-5A	No Fragmentase/half volume	25ul	G3	CAAGGTAC	1	P7_primer_Idx004	GCTACAAC	P5_primer_Idx010	ATCATGCG
11-5B	No Fragmentase/half volume	25ul	H3	GTCCTAAG	1	P7_primer_Idx004	GCTACAAC	P5_primer_Idx010	ATCATGCG
12-1A	No Fragmentase/half volume	25ul	A4	CCTCATCT	1	P7_primer_Idx004	GCTACAAC	P5_primer_Idx010	ATCATGCG
12-2B	No Fragmentase/half volume	25ul	B4	AATCCAGC	1	P7_primer_Idx004	GCTACAAC	P5_primer_Idx010	ATCATGCG
12-3A	No Fragmentase/half volume	25ul	C4	ACAGTTCG	1	P7_primer_Idx004	GCTACAAC	P5_primer_Idx010	ATCATGCG
12-3B	No Fragmentase/half volume	25ul	D4	CAACTCCA	1	P7_primer_Idx004	GCTACAAC	P5_primer_Idx010	ATCATGCG
12-4A	No Fragmentase/half volume	25ul	E4	CAATGCGA	1	P7_primer_Idx004	GCTACAAC	P5_primer_Idx010	ATCATGCG
12-4B	No Fragmentase/half volume	25ul	F4	GATGGAGT	1	P7_primer_Idx004	GCTACAAC	P5_primer_Idx010	ATCATGCG
13-1A	No Fragmentase/half volume	25ul	G4	TTCGAAGC	1	P7_primer_Idx004	GCTACAAC	P5_primer_Idx010	ATCATGCG
13-1B	No Fragmentase/half volume	25ul	H4	CCGAAGAT	1	P7_primer_Idx004	GCTACAAC	P5_primer_Idx010	ATCATGCG
13-2B	No Fragmentase/half volume	25ul	A5	ACTGCTTG	1	P7_primer_Idx004	GCTACAAC	P5_primer_Idx010	ATCATGCG
13-3A	No Fragmentase/half volume	25ul	B5	AGGTTCCT	1	P7_primer_Idx004	GCTACAAC	P5_primer_Idx010	ATCATGCG
13-3B	No Fragmentase/half volume	25ul	C5	ACGCAGTA	1	P7_primer_Idx004	GCTACAAC	P5_primer_Idx010	ATCATGCG
13-4A	No Fragmentase/half volume	25ul	D5	GGACTACT	1	P7_primer_Idx004	GCTACAAC	P5_primer_Idx010	ATCATGCG
13-4B	No Fragmentase/half volume	25ul	E5	GGCATTCT	1	P7_primer_Idx004	GCTACAAC	P5_primer_Idx010	ATCATGCG
10-1A	No Fragmentase/half volume	25ul	F5	CACAGACT	1	P7_primer_Idx004	GCTACAAC	P5_primer_Idx010	ATCATGCG
10-1B	No Fragmentase/half volume	25ul	G5	CGATTGGA	1	P7_primer_Idx004	GCTACAAC	P5_primer_Idx010	ATCATGCG
10-2A	No Fragmentase/half volume	25ul	H5	GACTTGTG	1	P7_primer_Idx004	GCTACAAC	P5_primer_Idx010	ATCATGCG
10-2B	No Fragmentase/half volume	25ul	A6	GAGGCATT	1	P7_primer_Idx004	GCTACAAC	P5_primer_Idx010	ATCATGCG
10-3A	No Fragmentase/half volume	25ul	B6	GCGTTAGA	1	P7_primer_Idx004	GCTACAAC	P5_primer_Idx010	ATCATGCG
10-3B	No Fragmentase/half volume	25ul	C6	ACGGACTT	1	P7_primer_Idx004	GCTACAAC	P5_primer_Idx010	ATCATGCG
10-5A	No Fragmentase/half volume	25ul	D6	ATTAGCCG	1	P7_primer_Idx004	GCTACAAC	P5_primer_Idx010	ATCATGCG
10-5B	No Fragmentase/half volume	25ul	E6	CACAGGAA	1	P7_primer_Idx004	GCTACAAC	P5_primer_Idx010	ATCATGCG
9-1B	No Fragmentase/half volume	25ul	F6	AGGAACAC	1	P7_primer_Idx004	GCTACAAC	P5_primer_Idx010	ATCATGCG
9-1A	No Fragmentase/half volume	25ul	G6	AGCCGTAA	1	P7_primer_Idx004	GCTACAAC	P5_primer_Idx010	ATCATGCG
9-2A	No Fragmentase/half volume	25ul	H6	ACTCTCCA	1	P7_primer_Idx004	GCTACAAC	P5_primer_Idx010	ATCATGCG
9-2B	No Fragmentase/half volume	25ul	A7	GTCCTTGA	1	P7_primer_Idx004	GCTACAAC	P5_primer_Idx010	ATCATGCG
9-3A	No Fragmentase/half volume	25ul	B7	TAGAACGC	1	P7_primer_Idx004	GCTACAAC	P5_primer_Idx010	ATCATGCG
9-3B	No Fragmentase/half volume	25ul	C7	CGTCAAGA	1	P7_primer_Idx004	GCTACAAC	P5_primer_Idx010	ATCATGCG
9-4A	No Fragmentase/half volume	25ul	D7	CTGCCATA	1	P7_primer_Idx004	GCTACAAC	P5_primer_Idx010	ATCATGCG
9-4B	No Fragmentase/half volume	25ul	E7	GGTGATGA	1	P7_primer_Idx004	GCTACAAC	P5_primer_Idx010	ATCATGCG
6-1A	No Fragmentase/half volume	25ul	F7	ACAGAGGT	1	P7_primer_Idx004	GCTACAAC	P5_primer_Idx010	ATCATGCG
6-1B	No Fragmentase/half volume	25ul	G7	CCTTCCAT	1	P7_primer_Idx004	GCTACAAC	P5_primer_Idx010	ATCATGCG
LIB_Neg_3	No Fragmentase/half volume	25ul	H7	CGAAGTCA	1	P7_primer_Idx004	GCTACAAC	P5_primer_Idx010	ATCATGCG
EBC-A1	No Fragmentase/half volume	25ul	A8	CGCAATGT	2	P7_primer_Idx004	GCTACAAC	P5_primer_Idx010	ATCATGCG
EBC-A2	No Fragmentase/half volume	25ul	B8	GAAGACTG	2	P7_primer_Idx004	GCTACAAC	P5_primer_Idx010	ATCATGCG
EBC-B2	No Fragmentase/half volume	25ul	C8	ACCATAGG	2	P7_primer_Idx004	GCTACAAC	P5_primer_Idx010	ATCATGCG
EBC010817	No Fragmentase/half volume	25ul	D8	CGGAGTAT	2	P7_primer_Idx004	GCTACAAC	P5_primer_Idx010	ATCATGCG
EBC1A	No Fragmentase/half volume	25ul	E8	AGCGTGTA	2	P7_primer_Idx004	GCTACAAC	P5_primer_Idx010	ATCATGCG
EBC1B	No Fragmentase/half volume	25ul	F8	TGCTGTGA	2	P7_primer_Idx004	GCTACAAC	P5_primer_Idx010	ATCATGCG
EBC2A	No Fragmentase/half volume	25ul	G8	TGGTATCC	2	P7_primer_Idx004	GCTACAAC	P5_primer_Idx010	ATCATGCG
EBC2B	No Fragmentase/half volume	25ul	H8	AACAGGTG	2	P7_primer_Idx004	GCTACAAC	P5_primer_Idx010	ATCATGCG
5-1A	No Fragmentase/half volume	25ul	A9	ATGGCGAT	2	P7_primer_Idx004	GCTACAAC	P5_primer_Idx010	ATCATGCG
5-1B	No Fragmentase/half volume	25ul	B9	AATGGTCG	2	P7_primer_Idx004	GCTACAAC	P5_primer_Idx010	ATCATGCG
5-2A	No Fragmentase/half volume	25ul	C9	GGTTGAAC	2	P7_primer_Idx004	GCTACAAC	P5_primer_Idx010	ATCATGCG
5-2B	No Fragmentase/half volume	25ul	D9	TCCTCATG	2	P7_primer_Idx004	GCTACAAC	P5_primer_Idx010	ATCATGCG
5-3A	No Fragmentase/half volume	25ul	E9	AGAACCAG	2	P7_primer_Idx004	GCTACAAC	P5_primer_Idx010	ATCATGCG
5-3B	No Fragmentase/half volume	25ul	F9	AACCGAAC	2	P7_primer_Idx004	GCTACAAC	P5_primer_Idx010	ATCATGCG
5-4A	No Fragmentase/half volume	25ul	G9	CTGTACCA	2	P7_primer_Idx004	GCTACAAC	P5_primer_Idx010	ATCATGCG
5-4B	No Fragmentase/half volume	25ul	H9	CTTACAGC	2	P7_primer_Idx004	GCTACAAC	P5_primer_Idx010	ATCATGCG
2-1A	No Fragmentase/half volume	25ul	A10	AACAAGGC	2	P7_primer_Idx004	GCTACAAC	P5_primer_Idx010	ATCATGCG
2-1B	No Fragmentase/half volume	25ul	B10	CCTTGGAA	2	P7_primer_Idx004	GCTACAAC	P5_primer_Idx010	ATCATGCG
2-2A	No Fragmentase/half volume	25ul	C10	AAGCTGGT	2	P7_primer_Idx004	GCTACAAC	P5_primer_Idx010	ATCATGCG
2-2B	No Fragmentase/half volume	25ul	D10	CCAAGTAG	2	P7_primer_Idx004	GCTACAAC	P5_primer_Idx010	ATCATGCG
16-1A	No Fragmentase/half volume	25ul	E10	GTACACCT	2	P7_primer_Idx004	GCTACAAC	P5_primer_Idx010	ATCATGCG
16-1B	No Fragmentase/half volume	25ul	F10	AAGACGAG	2	P7_primer_Idx004	GCTACAAC	P5_primer_Idx010	ATCATGCG
16-2A	No Fragmentase/half volume	25ul	G10	CGGCATTA	2	P7_primer_Idx004	GCTACAAC	P5_primer_Idx010	ATCATGCG
16-2B	No Fragmentase/half volume	25ul	H10	AATTCCGG	2	P7_primer_Idx004	GCTACAAC	P5_primer_Idx010	ATCATGCG
15-1A	No Fragmentase/half volume	25ul	A11	ATGCGTCA	2	P7_primer_Idx004	GCTACAAC	P5_primer_Idx010	ATCATGCG
15-1B	No Fragmentase/half volume	25ul	B11	AGCCTATC	2	P7_primer_Idx004	GCTACAAC	P5_primer_Idx010	ATCATGCG
15-2A	No Fragmentase/half volume	25ul	C11	CTCAGAAG	2	P7_primer_Idx004	GCTACAAC	P5_primer_Idx010	ATCATGCG
15-2B	No Fragmentase/half volume	25ul	D11	CTCCTAGT	2	P7_primer_Idx004	GCTACAAC	P5_primer_Idx010	ATCATGCG
14-1A	No Fragmentase/half volume	25ul	E11	GTAACCGA	2	P7_primer_Idx004	GCTACAAC	P5_primer_Idx010	ATCATGCG
14-1B	No Fragmentase/half volume	25ul	F11	CCGATGTA	2	P7_primer_Idx004	GCTACAAC	P5_primer_Idx010	ATCATGCG
14-2A	No Fragmentase/half volume	25ul	G11	GGAACATG	2	P7_primer_Idx004	GCTACAAC	P5_primer_Idx010	ATCATGCG
14-2B	No Fragmentase/half volume	25ul	H11	CCACATTG	2	P7_primer_Idx004	GCTACAAC	P5_primer_Idx010	ATCATGCG
14-3A	No Fragmentase/half volume	25ul	A12	TCTACGCA	2	P7_primer_Idx004	GCTACAAC	P5_primer_Idx010	ATCATGCG
14-3B	No Fragmentase/half volume	25ul	B12	CTCGTTCT	2	P7_primer_Idx004	GCTACAAC	P5_primer_Idx010	ATCATGCG
14-4A	No Fragmentase/half volume	25ul	C12	TCAGCCTT	2	P7_primer_Idx004	GCTACAAC	P5_primer_Idx010	ATCATGCG
14-4B	No Fragmentase/half volume	25ul	D12	CGAGTTAG	2	P7_primer_Idx004	GCTACAAC	P5_primer_Idx010	ATCATGCG
14-5A	No Fragmentase/half volume	25ul	E12	AGTCGAAG	2	P7_primer_Idx004	GCTACAAC	P5_primer_Idx010	ATCATGCG
14-5B	No Fragmentase/half volume	25ul	F12	CACTTCAC	2	P7_primer_Idx004	GCTACAAC	P5_primer_Idx010	ATCATGCG
LIB_NEG_4	No Fragmentase/half volume	25ul	G12	CCTACCTA	2	P7_primer_Idx004	GCTACAAC	P5_primer_Idx010	ATCATGCG
